# Air pollution and Alzheimer disease phenotype deplete esterified proresolving lipid mediator reserves in the brain

**DOI:** 10.1172/jci.insight.175917

**Published:** 2025-05-13

**Authors:** Ameer Y. Taha, Qing Shen, Yurika Otoki, Nuanyi Liang, Kelley T. Patten, Anthony E. Valenzuela, Christopher D. Wallis, Douglas J. Rowland, Abhijit J. Chaudhari, Keith J. Bein, Anthony S. Wexler, Lee-Way Jin, Brittany N. Dugger, Danielle J. Harvey, Pamela J. Lein

**Affiliations:** 1 Department of Food Science and Technology, College of Agriculture and Environmental Sciences,; 2 Center for Neuroscience, and; 3 West Coast Metabolomics Center, Genome Center, UCD, Davis, California, USA.; 4 Food and Biodynamic Laboratory, Graduate School of Agricultural Science, Tohoku University, Sendai, Miyagi, Japan.; 5 Department of Molecular Biosciences, School of Veterinary Medicine,; 6 Air Quality Research Center,; 7 Center for Genomic and Molecular Imaging, UCD, Davis, California, USA.; 8 Department of Radiology, School of Medicine, UCD, Sacramento, California, USA.; 9 Center for Health and the Environment and; 10 Departments of Mechanical and Aerospace Engineering, Civil and Environmental Engineering, and Land, Air and Water Resources, UCD, Davis, California, USA.; 11 Department of Pathology and Laboratory Medicine, UCD School of Medicine, Sacramento, California, USA.; 12 Department of Public Health Sciences, UCD, Davis, California, USA.; 13 The MIND Institute, School of Medicine, UCD, Sacramento, California, USA.

**Keywords:** Aging, Neuroscience, Alzheimer disease, Eicosanoids

## Abstract

**BACKGROUND:**

Traffic-related air pollution (TRAP) is a risk factor for Alzheimer disease (AD), where unresolved brain inflammation has been linked to deficits in the levels of free lipid mediators that enable the resolution of inflammation. It is unknown whether these deficits are due to reductions in esterified lipid pools, the main source of free bioactive proresolving lipids in the brain, and whether they are related AD pathophysiology.

**METHODS:**

This unknown was tested by measuring brain esterified lipid mediators and pathogenic markers of AD in TgF344-AD and WT male and female rats exposed to filtered air or TRAP for 14 months; it was also tested in human postmortem prefrontal cortex of individuals with or without AD.

**RESULTS:**

Significant reductions in proresolving lipid mediators esterified to neutral lipids and/or phospholipids were seen in AD and TRAP-exposed female rats, where levels were associated with inflammation, synaptic loss, and impaired glucose metabolism. Lower esterified proresolving lipid mediator concentrations were associated with older age in prefrontal cortex of humans with AD compared with controls.

**CONCLUSION:**

Impaired resolution in AD is due to depletion of esterified proresolving lipid pools that supply the brain with free bioactive mediators involved in inflammation resolution. TRAP exposure alters the same esterified resolution pathways, reflecting convergent mechanisms underlying AD.

## Abstract 2

In Alzheimer’s, there is a deficit in lipid reserves that supply the brain with pro-resolving lipid mediators. This may explain why inflammation is never resolved.

## Introduction

Alzheimer disease (AD), the main cause of age-related dementia, affects 6.2 million Americans aged 65 or older (1) and is the fifth-leading cause of death among the elderly (2). At present, there is no effective therapy for AD, which is why considerable efforts have been made to understand modifiable risk factors such as environmental exposures as well as etiologies underlying disease pathogenesis (3).

One environmental factor associated with increased risk of AD is chronic exposure to traffic-related air pollution (TRAP) (4, 5). TRAP is a complex and heterogeneous mixture of vehicle emissions, road dust, and secondary air pollutants including gases and particles (6). Evidence from epidemiological studies suggests that individuals who live less than 50 meters from a major roadway have a 7% increased risk of dementia compared with individuals living 200 meters away (7). Consistent with these observations, increased exposure to TRAP components including ozone, particulate matter 2.5, and/or nitrogen dioxide has been linked to a greater risk of AD (8, 9) or incident dementia (10).

Both AD and TRAP exposure are associated with immune activation characterized by an elevation in circulating and tissue (lung and brain) cytokines in rodents (11–14). In vivo, the effects of cytokines are mediated by short-lived bioactive lipid mediators (i.e., oxylipins) derived from the oxidation of polyunsaturated fatty acids via cyclooxygenase (COX) (15, 16), lipoxygenase (LOX) (17, 18), and cytochrome P450 (CYP) (19), or their degrading enzymes, which include 15-hydroxyprostaglandin dehydrogenase (15-PGDH) (20) and soluble epoxide hydrolase (sEH) (21–23). Proinflammatory oxylipins such as LOX-derived 12-hydroxyeicosatetraenoic acid (12-HETE) are elevated in the brain of transgenic animal models of AD (23, 24) and in postmortem brain of individuals with AD (17, 25, 26). Similarly, TRAP exposure has been shown to increase the concentration of proinflammatory oxylipins in human serum/plasma (e.g., LOX-derived 5-HETE) (27, 28).

Oxylipins are also involved in inflammation resolution, the process of halting inflammation, and repairing damaged cells (29). Resolution pathways are impaired in AD, as evidenced by a reported reduction of multiple proresolving oxylipins of docosahexaenoic acid (DHA), including 10R,17S-dihydroxydocosahexaenoic acid (neuroprotectin D1), maresin 1, and of arachidonic acid (AA) (e.g., lipoxin A4 [LXA4]), in cerebrospinal fluid, hippocampus, prefrontal cortex, or entorhinal cortex of patients with AD compared with patients without AD (non-AD controls) (26, 30–32). Consistent with human studies, brain concentrations of proresolving DHA-derived neuroprotectin D1 and epoxides (epoxydocosapentaenoic acids [EpDPEs]), eicosapentaenoic acid–derived (EPA-derived) hydroxides (resolvin E1 and hydroxyeicosapentaenoic acid [HEPE]), and AA-derived epoxides (epoxyeicosatrienoic acids [EpETrEs]) and lipoxins were shown to be reduced in transgenic mouse models of AD compared with genetically unaltered controls (21, 23, 24, 33, 35–38). AA-derived 15-HETE, a precursor to trihydroxylated proresolving lipoxins (39) was also reported to decrease in the brain of transgenic AD mice compared with controls (24), and in cerebral cortex of individuals with AD compared with those without AD (40). It is not known whether TRAP exposure, a risk factor for AD, alters the same inflammation resolution oxylipin pathways in the brain.

To date, all studies have characterized lipid mediator disturbances in AD by measuring the concentration of free (i.e., unesterified) oxylipins. Although oxylipins are enzymatically synthesized in the brain by COX, LOX, 15-PGDH, CYP450, and sEH, they can also be released from or sequestered (i.e., reesterified) into the more abundant esterified lipid pool as shown in the pathway illustrated in Supplemental Figure 1 (supplemental material available online with this article; https://doi.org/10.1172/jci.insight.175917DS1). In this regard, we reported that approximately 90% of oxylipins in the rat and human brain are bound to phospholipids (PL) and neutral lipids (NL) consisting of triacylglycerides and cholesteryl esters (41–43). We also showed, in vivo, that esterified oxylipins can both release or sequester free oxylipins through a turnover pathway that regulates the bioavailability of the free oxylipin pool (42, 44). Free oxylipins are bioactive (45, 46), whereas oxylipins bound to PL and NL are relatively inactive (47, 48).

Although the evidence in rodent models of AD and in humans with AD point to reductions in brain free proresolving oxylipins (36, 37), it is not known whether esterified oxylipins involved in providing free oxylipins that resolve inflammation are altered in AD or by TRAP exposure, or whether they relate to markers of AD pathogenesis including inflammation (49, 50), synaptic loss (51, 52), and brain energy deficits linked to neuronal/synaptic loss (53). This knowledge gap is important to address because changes in the esterified oxylipin pool may mechanistically explain why free proresolving oxylipins are reduced in AD. Indeed, based on the evidence to date, reductions in free proresolving oxylipins characterized in AD could not be attributed to their synthetic enzymes (12/15-LOX, 5-LOX, and sEH), as these enzymes were shown to increase (rather than decrease) in both rodent models of AD (23, 34) and in human AD postmortem brain (17, 18).

The purpose of this study was 4-fold. First, we tested whether bound (i.e., esterified) oxylipins involved in inflammation and inflammation resolution are altered in transgenic AD rats expressing mutations in the human Swedish amyloid precursor protein (APPswe) and Δ exon 9 mutant presenilin-1 (PS1ΔE9), that were exposed to filtered air (FA) or chronic TRAP, a significant risk factor for AD pathogenesis. Second, we explored whether changes in esterified oxylipins are linked to aspects of AD pathogenesis including PET imaging of translocator protein (TSPO), synaptic vesicle glycoprotein 2A (SV2A), and glucose uptake, as markers of neuroinflammation (54), synaptic loss (51), and glucose metabolism (55), respectively. Third, because the incidence of AD is greater in females than males (56, 57), we explored whether females would be more affected than males by the effects of AD and TRAP exposure. Fourth, we aimed to establish translational relevance by testing whether disturbances in esterified oxylipin metabolism occur in the postmortem prefrontal cortex of individuals with AD compared with those without AD. 

## Results

### Effects of AD genotype and TRAP exposure on NL-bound oxylipins in brain of 15-month-old rats.

**Figure 1:**
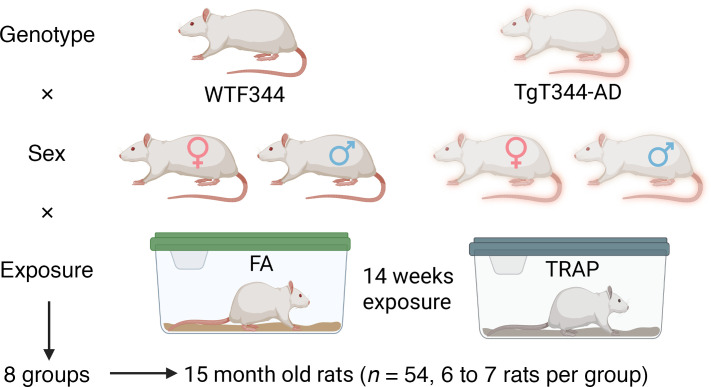
Overall study design. Male and female rats were exposed to filtered air (FA) or traffic-related air pollution (TRAP) from 1 to 15 months of age. After euthanasia, the brains were subjected to lipidomic analysis of neutral lipid (NL) and phospholipid (PL) bound oxylipins.

In order to assess the effects of AD genotype or TRAP exposure on NL-bound oxylipins, rats expressing mutations in APPswe and PS1ΔE9, henceforth called AD transgenic rats or WT rats, were exposed to FA or TRAP from 1 to 15 months of age as shown in Figure 1. Esterified oxylipins were captured using the mass spectrometry (MS) metrics shown in Supplemental Table 1 and imputation parameters for missing values shown in Supplemental Table 2.

**Figure 2:**
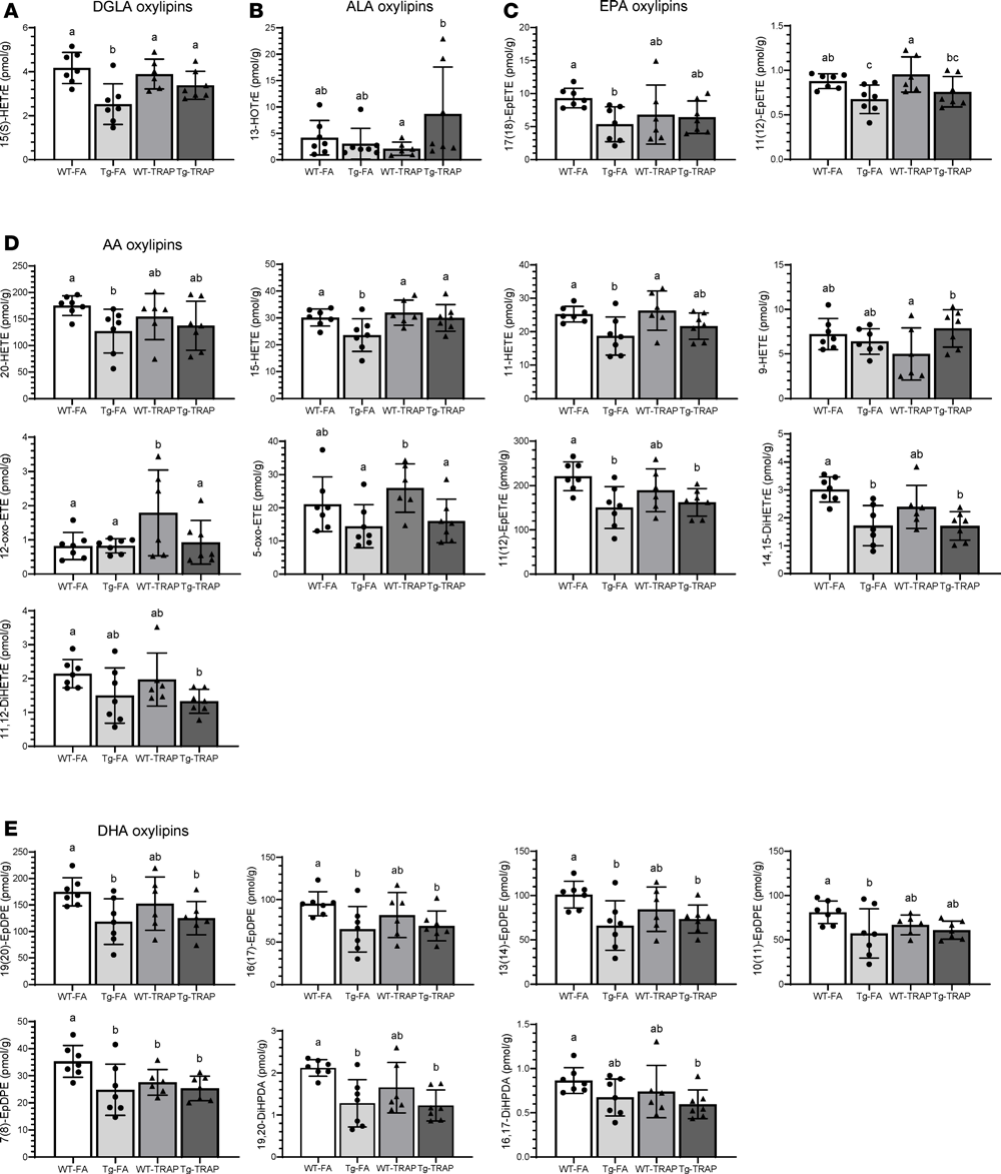
Oxylipin concentrations in brain NL of 15-month-old WT or TgF344-AD (Tg) female rats exposed to FA or TRAP for 14 months. Data are shown as mean ± SD of *n* = 7 WT-FA, *n* = 7 Tg-FA, *n* = 6 WT-TRAP, and *n* = 7 Tg-TRAP. Data were analyzed by 1-way ANOVA followed by Duncan’s post hoc test. Different-letter superscripts indicate that the means differed significantly from each other at *P* < 0.05. (**A**–**E**) DGLA-derived oxylipins (**A**), ALA-derived oxylipins (**B**), EPA-derived oxylipins (**C**), AA-derived oxylipins (**D**), and DHA-derived oxylipins (**E**). DiHETE, dihydroxyeicosatetraenoic acid; DiHETrE, dihydroxyeicosatrienoic acid; DiHOME, dihydroxyoctadecenoic acid; DiHDPA, dihydroxydocosapentaenoic acid; EpDPE, epoxydocosapentaenoic acid; EpETE, epoxyeicosatetraenoic acid; EpETrE, epoxyeicosatrienoic acid; EpOME, epoxyoctadecenoic acid; HDoHE, hydroxydocosahexaenoic acid; HEPE, hydroxyeicosapentaenoic acid; HETE, hydroxyeicosatetraenoic acid; HETrE, hydroxyeicosatrienoic acid; HODE, hydroxyoctadecadienoic acid; HOTrE, hydroxyoctadecatrienoic acid; oxo-ETE, oxo-eicosatetraenoic acid; oxo-ODE, oxo-octadecadienoic acid; TriHOME, trihydroxyoctadecenoic acid.

AD transgenic rats showed significant changes in NL-bound oxylipins in females but not males (*P* < 0.05 for main effects of sex and genotype; Supplemental Table 3). As shown in Figure 2, compared with WT female rats, significant reductions in NL-bound oxylipin were observed in TgF344-AD female rats exposed to either FA or TRAP (*P* < 0.05 by 1-way ANOVA), whereas only a few significant changes were detected in males (Supplemental Table 4; *P* < 0.05 by 1-way ANOVA).

Most of the changes in females were observed in oxylipins involved in inflammation resolution (e.g., EpETrEs, EpETEs, and EpDPEs) or oxylipins destined toward proresolving lipid mediator synthesis (e.g., HETE precursors to lipoxins). As shown in Figure 2, dihomo-γ-linoleic acid–derived (DGLA-derived) 15(S)-HETrE (Figure 2A); EPA-derived 17(18)-EpETE and 11(12)-EpETE (Figure 2C); AA-derived 20-HETE, 15-HETE, 11-HETE, 11(12)-EpETrE, and 14,15-DiHETrE (Figure 2D); and DHA-derived 19(20)-EpDPE, 16(17)-EpDPE, 13(14)-EpDPE, 10(11)-EpDPE, 7(8)-EpDPE, and 19,20-DiHDPA (Figure 2E) were significantly lower by 22%–43% in Tg-FA rats compared with WT-FA controls (*P* < 0.05). The majority of these oxylipins [AA-derived 11(12)-EpETrE and 14,15-DiHETrE, and DHA-derived 19(20)-EpDPE, 16(17)-EpDPE, 13(14)-EpDPE, 7(8)-EpDPE, and 19,20-DiHDPA], as well as AA-derived 5-oxo-ETE and 11,12-DiHETrE, and DHA-derived 16,17-DiHDPA, were also lower by 8%–43% in Tg-TRAP exposed rats compared with WT-FA or WT-TRAP, suggesting an AD-effect, independent of TRAP exposure. α-Linolenic acid–derived (ALA-derived) 13-hydroxyoctadecatrienoic acid (13-HOTrE) was 4-fold higher in Tg-TRAP compared with WT-TRAP rats (Figure 2B; *P* < 0.05), but neither groups differed significantly from WT-FA controls.

TRAP exposure alone minimally affected NL oxylipins in WT rats. The few observed changes included a significant increase in AA-derived 12-oxo-ETE by ~2-fold in WT-TRAP rats compared with WT-FA, Tg-FA, and Tg-TRAP rats (Figure 2D), and there was a significant 22% decrease in DHA-derived 7(8)-EpDPE in WT-TRAP rats compared with WT-FA controls (Figure 2E).

Overall, the data suggest that the AD genotype reduced multiple proresolving oxylipins in NL of female rats. Of the ~17 significantly altered oxylipins in TgF344-AD rats exposed to FA or TRAP, 11 (65%) have proresolving effects in vivo, 2 (11-HETE, 20-HETE) have proinflammatory effects, and 4 are sEH inactivation products of fatty acid epoxides (11,12-DiHETrE, 14,15-DiHETrE, 16,17-DiHDPA, and 19,20-DiHDPA).

### Effects of AD genotype and TRAP exposure on PL-bound oxylipins in brain of 15-month-old rats.

**Figure 3:**
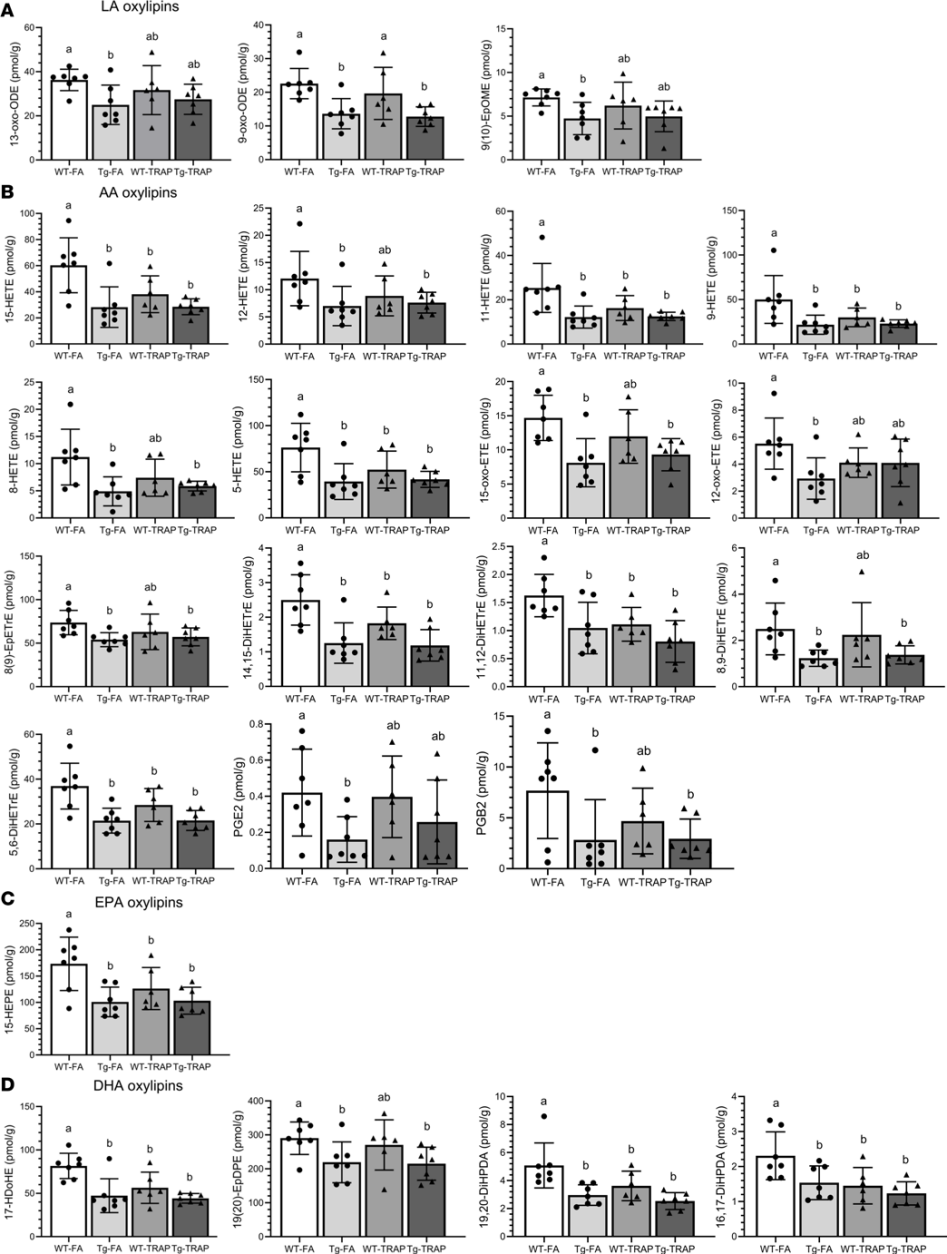
Oxylipin concentrations in brain PL of 15-month-old WT or TgF344-AD (Tg) female rats exposed to FA or TRAP for 14 months. Data are shown as mean ± SD of *n* = 7 WT-FA, *n* = 7 Tg-FA, *n* = 6 WT-TRAP, and *n* = 7 Tg-TRAP. Data were analyzed by 1-way ANOVA followed by Duncan’s post hoc test. Different-letter superscripts indicate that the means differed significantly from each other at *P* < 0.05. (**A**–**D**) LA-derived oxylipins (**A**), AA-derived oxylipins (**B**), EPA-dedrived oxylipins (**C**), and DHA-derived oxylipins (**D**). DiHETE, dihydroxyeicosatetraenoic acid; DiHETrE, dihydroxyeicosatrienoic acid; DiHOME, dihydroxyoctadecenoic acid; DiHDPA, dihydroxydocosapentaenoic acid; EpDPE, epoxydocosapentaenoic acid; EpETE, epoxyeicosatetraenoic acid; EpETrE, epoxyeicosatrienoic acid; EpOME, epoxyoctadecenoic acid; HDoHE, hydroxydocosahexaenoic acid; HEPE, hydroxyeicosapentaenoic acid; HETE, hydroxyeicosatetraenoic acid; HETrE, hydroxyeicosatrienoic acid; HODE, hydroxyoctadecadienoic acid; HOTrE, hydroxyoctadecatrienoic acid; oxo-ETE, oxo-eicosatetraenoic acid; oxo-ODE, oxo-octadecadienoic acid; TriHOME, trihydroxyoctadecenoic acid; PG, prostaglandin.

We next examined the effects of AD or TRAP exposure on PL-bound oxylipins. There were no significant effects of AD genotype or TRAP exposure in males (Supplemental Table 5). However, significant changes due to genotype or TRAP were observed in females mainly (Supplemental Table 6) as depicted in Figure 3.

AD genotype significantly reduced linoleic acid– (LA-), AA-, EPA-, and DHA-derived oxylipins. Compared with WT-FA controls, the Tg-FA group exhibited a significant reduction in LA-derived 13-oxo-ODE, 9-oxo-ODE, and 9(10)-EpOME (by 31%–40%; Figure 3A); AA-derived 5-,8-.9-,11-,12- and 15-HETEs; 12- and 15-oxo-ETE; 8(9)-EpETrE; 5,6-DiHETrEs, 8,9-DiHETrEs, 11,12-DiHETrEs, and 14,15-DiHETrEs; PGE2, and PGB2 (by 27%–63%; Figure 3B); EPA-derived 15-HEPE (by ~42%; Figure 3C); and DHA-derived 17-hydroxydocosahexaenoic acid (17-HDoHE), 19(20)-EpDPE, 19,20-DiHDPA, and 16,17-DiHDPA (by 24%–42%; Figure 3D). Similar significant reductions in PL-bound oxylipins were observed in Tg-TRAP rats compared with WT-FA controls.

TRAP exposure alone resulted in similar reductions in AA-, EPA-, and DHA-derived PL-bound oxylipins in WT rats. Compared with WT-FA controls, WT-TRAP rats showed significant reductions in AA-derived 15-HETE, 11-HETE, 9-HETE, 5-HETE, 14,15-DiHETrE, 11,12-DiHETrE, and 5,6-DiHETrE by 23%–40% (Figure 3B; *P* < 0.05); EPA-derived 15-HEPE by 27% (Figure 3C; *P* < 0.05); and DHA-derived 17-HDoHE, 19,20-DiHDPA, and 16,17-DiHDPA by 29% to 37 % (Figure 3D; *P* < 0.05).

Of the 23 significantly altered oxylipin in PL by AD genotype and/or TRAP exposure, 7 (30%) are proresolving or serve as precursors to proresolving mediators [5-HETE, 15-HETE, 12-oxo-ETE, 15-oxo-ETE, 8(9)-EpETrE, 15-HEPE, 17-HDoHE], 8 are proinflammatory, 6 are diols of sEH epoxide metabolism, and 2 are prostanoids.

### Association between rat brain in vivo imaging measures of AD markers of pathophysiology and esterified oxylipins.

**Figure 4:**
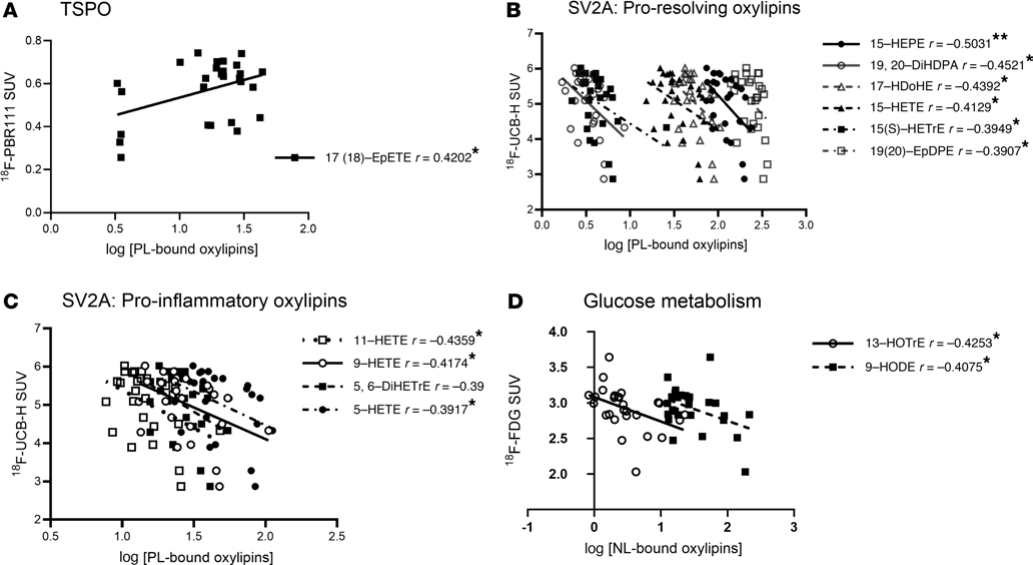
Pearson’s correlation between brain in vivo imaging markers of AD pathology and brain esterified oxylipins in female rats. The figures show linear plots and significant Pearson’s correlation coefficient *r* values between in vivo imaging markers of AD pathogenesis and log oxylipin concentrations within PL or NL in female rats (*n* = 27). (**A**) ^18^F-PBR111 average whole brain SUV and log-transformed PL-bound oxylipins. (**B**) ^18^F-UCB-H average whole brain SUV and log-transformed proresolving PL-bound oxylipins. (**C**) ^18^F-UCB-H average whole brain SUV and log-transformed proinflammatory PL-bound oxylipins. (**D**) ^18^F-FDG average whole brain SUV and log-transformed NL-bound oxylipins. *P < 0.05 and **P < 0.01. DiHETrE, dihydroxyeicosatrienoic acid; DiHDPA, dihydroxydocosapentaenoic acid; EpDPE, epoxydocosapentaenoic acid; EpETE, epoxyeicosatetraenoic acid; EpETrE, epoxyeicosatrienoic acid; HDoHE, hydroxydocosahexaenoic acid; HEPE, hydroxyeicosapentaenoic acid; HETE, hydroxyeicosatetraenoic acid; HETrE, hydroxyeicosatrienoic acid; HODE, hydroxyoctadecadienoic acid; HOTrE, hydroxyoctadecatrienoic acid.

We next explored the relationship between brain esterified oxylipins and markers of AD neuropathology (TSPO, SV2A, and fluorodeoxyglucose) in females. There were no significant group differences between the groups in MRI measures of brain atrophy or PET uptake values for tracers targeted toward TSPO (marker of neuroinflammation), SV2A (marker of synaptic density), and fluorodeoxyglucose (marker of glucose metabolism) in 15-month-old female rats (Table 1). PL-bound 17(18)-EpETE (from EPA) was positively associated with the standardized uptake value (SUV) of the TSPO-targeted radiotracer as shown in Figure 4A (*P* = 0.029, *r* = 0.4202), meaning more sequestration of this proresolving lipid mediator was linked to more inflammation. Several PL-bound oxylipins were negatively correlated with the SUV of the SV2A-targeted radiotracer, including 6 proresolving oxylipins (Figure 4B) and 4 proinflammatory oxylipins (Figure 4C). The proresolving oxylipins were DHA-derived 17-HDoHE, 19,20-DiHPDA, and 19(20)-EpDPE; EPA-derived 15-HEPE; AA-derived 15-HETE; and ALA-derived 15(S)-HETrE (*r* =–0.5031 to –0.3907; *P* = 0.008–0.044). The proinflammatory oxylipins were AA-derived 11-HETE, 9-HETE, 5-HETE, and 5,6-DiHETrE (*r* = –0.4359 to –0.3917, *P* = 0.023 to 0.043). This means that the presence of more of these oxylipin in the esterified PL form was associated with fewer synapses.

There were no significant correlations between NL-bound oxylipins and SUV of the TSPO- and SV2A-targeted radiotracers. However, ALA-derived 13-HOTrE (*P* = 0.027, *r* = –0.4253) and LA-derived 9-hydroxyoctadecadienoic acid (9-HODE) (*P* = 0.035, *r* = –0.4075) in NL were negatively correlated with ^18^F-FDG SUV (Figure 4D), meaning greater esterified levels of these compounds were related to lower brain glucose metabolism.

### Association between age at death and esterified oxylipins in human postmortem brains.

We next explored whether the observed link between brain esterified oxylipins and markers of AD pathogenesis in rats translated to human postmortem prefrontal cortex of individuals with or without AD.

**Figure 5:**
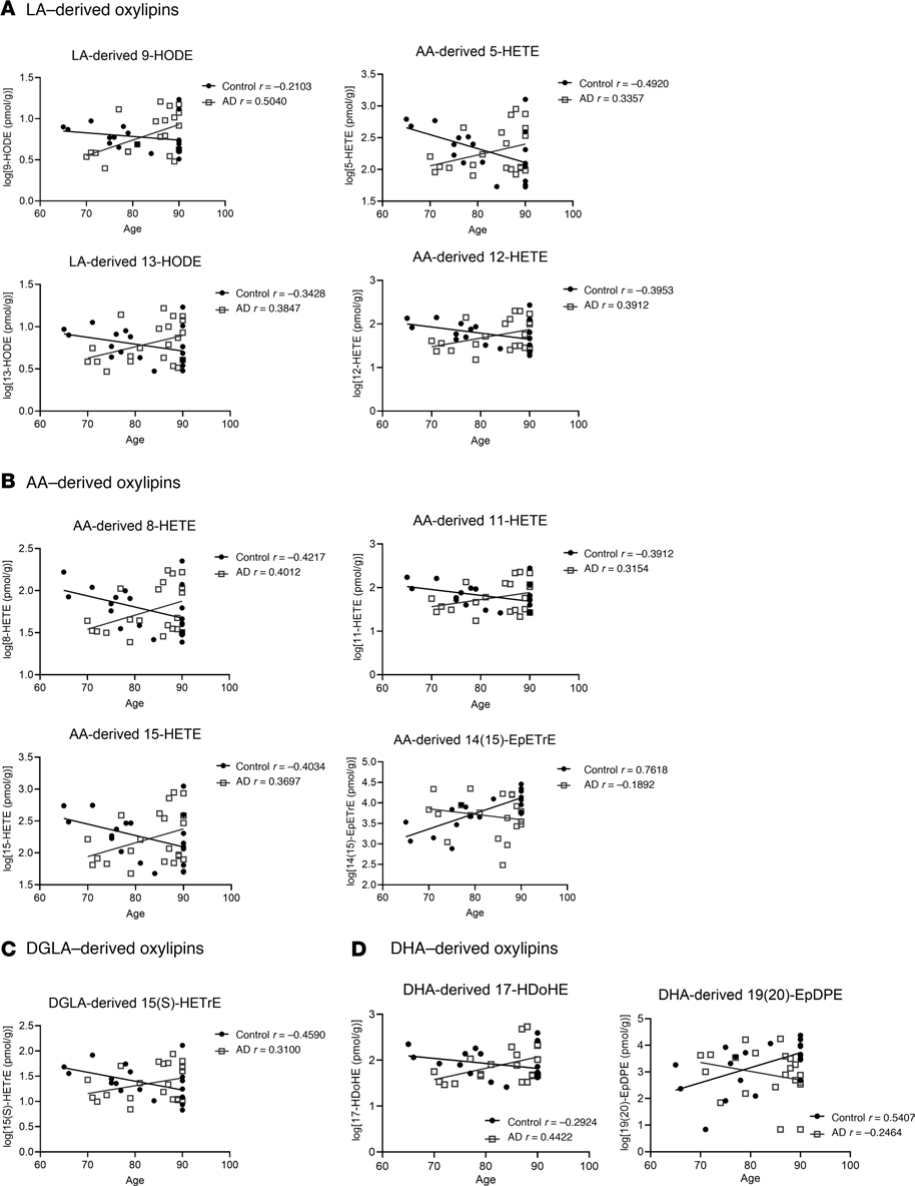
Association between PL-bound oxylipins (log-transformed values) and age in prefrontal cortex of individuals with AD and without AD. Linear plots and Pearson’s correlation coefficient (*r* value) between oxylipins and age in the Control (*n* = 20) and AD (*n* = 21) groups. These plots are used to visualize associations that reached statistical significance by multiple linear regression analysis applied on log-transformed concentrations of each oxylipin as the dependent variable, and group, sex, mean-centered postmortem interval (PMI), and mean centered age were tested for main effects (independent variables). Interaction effects were tested between group and age as well as between group and sex. (**A**–**D**) LA-derived oxylipins (**A**), DGLA-derived oxylipins (**B**), AA-derived oxylipins (**C**), and DHA-derived oxylipins (**D**). *r* values in the figure represent Pearson’s correlation coefficient of the linear plot correlating log-transformed oxylipins to age for each of the AD and non-AD groups. EpDPE, epoxydocosapentaenoic acid; EpETE, epoxyeicosatetraenoic acid; EpETrE, epoxyeicosatrienoic acid; HDoHE, hydroxydocosahexaenoic acid; HETE, hydroxyeicosatetraenoic acid; HETrE, hydroxyeicosatrienoic acid; HODE, hydroxyoctadecadienoic acid.

There were no significant associations between oxylipins and AD in NL. However, a significant age/AD interaction was observed in 11 oxylipins esterified to PL, after adjusting for postmortem interval (PMI), sex, and age at death (Table 2 and Supplemental Table 7). As shown in Figure 5, proresolving epoxides of AA [14(15)-EpETrE] (Figure 5B) and DHA [19(20)-EpDPE] (Figure 5D) decreased with age at death in individuals with AD and increased with age at death in non-AD controls. With increasing age at death, 11 PL-bound oxylipins of LOX enzymatic synthesis increased in AD but decreased in the control (non-AD) group. These included LA-derived 9-HODE and 13-HODE (Figure 5A); DGLA-derived 15(S)-HETrE (Figure 5C); AA-derived 5-HETE, 8-HETE, 11-HETE, 12-HETE, and 15-HETE (Figure 5B); and DHA-derived 17-HDoHE (Figure 5D), of which 17-HDoHE, 5-HETE, and 15-HETE are precursors to proresolving lipid mediators not detected in this study, while the other compounds are proinflammatory.

## Discussion

The main finding of this study is that, in both rats and humans with AD pathology, there was a significant depletion of proresolving esterified oxylipins that provide the brain with free oxylipins involved in mediating the resolution of inflammation. In rats, this depletion was also caused by chronic TRAP exposure, a risk factor for AD, and was associated with PET markers of neuroinflammation, synaptic loss, and impaired glucose metabolism. In humans, reductions in several proresolving esterified oxylipins were observed with age at death in the AD group only, further corroborating the rat findings.

Prior studies have consistently shown a reduction in free proresolving lipid mediators (i.e., oxylipins) in the brain of transgenic mouse models of AD (e.g., resolvins, EpDPEs, EpETrEs, and lipoxins; refs. 21, 23, 30, 35) and in the postmortem brain of individuals with AD; e.g., resolvin D5, maresin 1 and protectin D1 (26, 30), and LXA4 (58). However, the mechanism underlying these observations have never been established. Changes in LOX and CYP synthetic enzymes do not account for the reported reductions in free proresolving lipids, as postmortem rodent and human studies have shown that these enzymes are in fact elevated in AD (23, 34). Our findings show that the source of these free bioactive proresolving oxylipins (i.e., the esterified pool) is markedly depleted in AD, providing a mechanistic explanation for why free proresolving mediators are reduced in AD. In this study, we also extend these findings to TRAP exposure, a significant risk factor for AD dementia (4, 5).

The effects of AD pathophysiology on esterified proresolving lipid mediators were age dependent. Previously, we reported significant reductions in 4 esterified proresolving oxylipins in 10-month-old TgF344-AD rats (59), prior to the onset of cognitive impairment, neuroinflammation, and plaque and phosphorylated tau accumulation (60). These changes were exacerbated in the present study, where 18 proresolving lipids within NL and PL decreased by 15 months of age in TgF344-AD rats, when cognitive impairment, neuroinflammation, and AD pathogenic markers were established (60). In humans with AD pathology, older age was associated with greater reductions in esterified proresolving oxylipins [14(15)-EpETrE] and DHA [19(20)-EpDPE], consistent with the age-dependent changes observed in AD rats. Overall, both the rat and human data point to age-dependent impairments in the brain’s capacity to resolve neuroinflammation due to progressive depletion of esterified lipid reserves that supply the brain with free proresolving oxylipins.

Changes in brain lipid mediator turnover involving the release of bound oxylipins and reesterification of free oxylipins likely explain the observed reductions in esterified lipid mediators. Oxylipin release is enabled by lipase enzymes (61), whereas reesterification is catalyzed by the acylation of free oxylipins via fatty acyl-CoA synthetases (62) and the subsequent esterification of acylated oxylipins (i.e., oxylipin-CoA) into NL and PL by acyltransferases (e.g., sn-glycerol-3-phosphate acyltransferases/lysophosphatidic acid acyltransferases) (63). Acyl-Co synthetase 4 preferentially incorporates AA-derived epoxides into PL (61), potentially implicating this particular isoform in the observed reduction in PL-bound AA-epoxides in AD (rats and humans); however, the specific lipase and acyltransferase isoforms potentially involved in brain proresolving oxylipin turnover are not known.

TRAP exposure reduced esterified oxylipin concentrations in the brains of WT female rats similar to what we observed in TgF344-AD female rats, suggesting that both environmental and genetic predispositions to AD target the same lipid esterification pathways. A notable distinction, however, is that AD genotype reduced proresolving lipids in both NL and PL, whereas TRAP exposure reduced them almost exclusively within PL. It is not entirely clear why different lipid pools were affected by the 2 conditions, when neuroinflammation plays a central role in both AD and TRAP exposure. It is possible that NL-bound oxylipins might change with prolonged exposure to TRAP in rats.

In humans, mainly PL were affected by AD pathology, suggesting that PL-bound oxylipins are more vulnerable than NL-bound oxylipins to the effects of AD pathogenesis. As in rats with AD (59), NL-bound oxylipins might change with time in humans, if the duration of AD pathogenesis was longer. It is possible that the brain utilizes PL-bound oxylipins first before utilizing NL-bound oxylipins toward generating free proresolving lipid mediators. This remains to be confirmed in human studies that account for the duration of AD before death.

In general, there were no additive effects between AD genotype and TRAP exposure in rats, meaning that TRAP exposure did not further exacerbate the deficits in esterified oxylipin concentrations in AD transgenic rats, compared with FA exposure. This could be because both AD genes and TRAP act on a common target (e.g., enzyme or receptor) that release or reesterify free oxylipins. The net effect, as observed in this study, is a reduction in esterified proresolving oxylipins by either AD genotype or TRAP exposure.

In rats, several esterified oxylipins, particularly proresolving oxylipins, were associated with PET imaging measures of neuroinflammation, synaptic loss, and metabolic deficits (Figure 5). The PET measure from the TSPO-targeted radiotracer assessing neuroinflammation was positively correlated with proresolving 17(18)-EpETE in PL. Increased esterification of 17(18)-EpETE reflects reduced turnover and availability of this proresolving lipid mediator in the free bioactive form, which could exacerbate inflammation previously documented in AD and TRAP-exposed rats (14). In contrast, the SUV measure from the SV2A-targeted radiotracer was inversely correlated with PL-bound proresolving oxylipins. A higher SUV likely reflects increased number of binding sites in response to atrophy and neuronal cell loss, particularly in TRAP-exposed or TgF344-AD rats (60, 64). Thus, the observed inverse association between SV2A-targeted radiotracer and PL-bound proresolving oxylipins reflect reduced turnover and availability of proresolving oxylipins in the free form, which could accelerate synaptic loss.

Two NL-bound oxylipins (9-HODE and 13-HOTrE) were inversely correlated with uptake of ^18^F-FDG (a marker of brain glucose metabolism; Figure 5D), indicating that higher concentrations of these bound oxylipins is linked to impaired glucose utilization. In AD, brain glucose utilization decreases (65–67), both due to neuronal/synaptic loss and mitochondrial dysfunction, leading to energy deficits (53, 67, 68). We are not aware of any literature on the effects of esterified or free 9-HODE and 13-HOTrE on brain energy metabolism, although both oxylipins in the free form were reported to regulate adipocyte metabolism (69), suggesting their potential role in energy regulation. Further studies are needed to explore this newly identified association between bound oxylipins and brain energy metabolism.

Although proresolving esterified oxylipins decreased in both rats (AD and TRAP-exposed) and humans (with AD), several proinflammatory oxylipins did not follow a consistent trend when comparing rats to humans. In rats, LA-derived 9-oxo-ODE, 13-oxo-ODE, and 9(10)-EpOME and AA-derived DiHETrEs were reduced by 31%–51% in PL of female TgF344-AD rats as were AA-derived HETEs, which were reduced by 26%–57% in both NL and PL of female TgF344-AD rats and TRAP-exposed WT rats. In humans, however, age was associated with increased levels of many of these oxylpins within PL of patients with AD. In rats, the observed reduction in proinflammatory mediators may reflect increased lipase-mediated release in response to proinflammatory cytokines shown to be elevated in the brain of AD transgenic and TRAP-exposed rats (14). This would be consistent with the observed inverse association between ^18^F-UCB-H uptake, reflecting increased numbers of synaptic binding sites due to neuronal loss, and 4 proinflammatory PL-bound oxylipins (i.e., more synaptic loss seems to be linked to increased hydrolysis of esterified proinflammatory oxylipins). In humans, such deficits may occur with disease advancement. Thus, the fact that the esterified proinflammatory lipids were increased with age in individuals with AD likely reflects protective mechanisms that esterify these inflammatory lipid mediators to slow disease progression. Over time, these mechanisms may fail, leading to further neurodegeneration, similar to what we observed in TgF344-AD rats (14, 59, 60).

The effects of AD-genotype and TRAP were mainly seen in female rats, suggesting greater vulnerability of females to AD and TRAP exposure. This is consistent with epidemiological data showing that the incidence of AD is about twice greater in women than in men (56, 70). TRAP exposure may also contribute to sex vulnerabilities to dementia, as a recent study found that, compared with men, women had a significantly higher risk for cognitive decline associated with increased exposure to air pollution (i.e., PM_10_, PM_2.5−10_, and NO_2_) (71). This is mechanistically aligned with findings of this study showing sex-specific changes in esterified lipid mediators and with our previous study showing that TRAP-exposed females had more amyloid plaque deposition compared with TRAP-exposed males at early ages (60). We did not observe sex-specific effects in humans, likely due to the low number of female participants in the study.

One limitation of this study is that the animals were moved from the exposure tunnel to the UCD main campus vivarium for 23 days (for MRI and PET imaging) prior to euthanasia. This exposure-free period is unlikely to change TRAP-related outcomes, as it is known that PM and various dust elements accumulate and reside in the brain for a few months after exposure (72–75). Another limitation is that we only measured brain esterified oxylipins and imaging markers of AD pathogenesis in rat whole brain and in human prefrontal cortex. Future studies should investigate the involvement of different brain cell types (glia or neurons) across various brain regions known to be involved in AD pathogenesis (e.g., entorhinal cortex, hippocampus) and address whether the changes we saw are driven by resident brain cells or infiltrating immune cells (36, 76). Finally, information on the duration of AD pathology in humans is lacking, making it difficult to track how different oxylipin pools change with disease advancement.

In summary, our study provides evidence of disturbances in esterified oxylipin pools involved in regulating the availability of free proresolving lipid mediators in the brain of rats and humans with AD pathology. We show, for the first time to our knowledge, that chronic TRAP exposure targets the same lipid network implicated in AD. Collectively, our findings may explain why inflammation resolution pathways are impaired in AD and why chronic TRAP exposure increases the risk of AD type dementia (i.e., by impairing resolution pathways involving esterified lipids). Targeting proresolving lipid mediators release or esterification may have therapeutic benefits in AD caused by genetic vulnerabilities or chronic TRAP exposure.

## Methods

### Sex as a biological variable.

Our study was performed in rats to explore the effects of AD genotype and TRAP exposure on brain esterified lipid meditators, and it was performed in human postmortem brain samples to establish translational relevance of the rodent findings. Our study examined both male and female rats, and sex-dimorphic effects are reported. Although both male and female postmortem brains were measured, we were not statistically powered to explore sex-specific effects in humans, and therefore male and female data were grouped.

### Chemicals and reagents.

Ethylenediaminetetraacetic acid (EDTA; catalog EDS-100G), butylated hydroxytoluene (BHT; catalog W218405-SAMPLE-K), and triphenyl phosphine (TPP; catalog 3T84409) were purchased from Sigma-Aldrich.

Oxylipin standards were purchased from Cayman Chemical or Loradan Biomedical. Deuterated surrogate standards used for oxylipin quantitation were obtained from Cayman Chemical. These include d11-11(12)-EpETrE (catalog 10006413), d11-14,15-DiHETrE (catalog 1008040), d4-6-keto-PGF1α (catalog 315210), d4-9-HODE (catalog 338410), d4-LTB4 (catalog 320110), d4-PGE2 (catalog 314010), d4-TXB2 (catalog 319030), d6-20-HETE (catalog 390030), and d8-5-HETE (catalog 334230).

### Animals and TRAP exposure.

Male TgF344-AD transgenic rats expressing human APPsw and PS1ΔE9 genes were obtained from Emory University (Atlanta, Georgia, USA) (57). Female WT Fischer 344 (WTF344) rats were purchased from Charles River Laboratories. Male TgF344-AD and female WTF344 rats were bred by the Lein laboratory at UCD, and the resulting offspring were genotyped as described (60). On P28 (approximately 1 month of age), 54 rats (27 males and 27 females consisting of TgF344-AD and WTF344 rats each) were transferred to a tunnel facility situated near a heavily trafficked freeway tunnel system in Northern California, USA (77). Half of the rats per genotype and per sex were randomly assigned to the FA versus TRAP groups and exposed continuously for up to 14 months as previously described (78). Thus, there were 8 groups in total as shown in the overall study design depicted in Figure 2 (*n* = 54 rats in total, 8 groups, 6 or 7 rats per group). The animals were euthanized at 15 months of age.

The tunnel facility was built to capture gaseous and particulate components of real-world TRAP (77). It had a filtering system that provided FA to exposure chambers housing the FA groups or TRAP collected from the traffic tunnel and delivered unchanged in real-time to exposure chambers housing the TRAP groups. During the 14-month–long exposure period, total particle numbers and mean 24-hour PM_2.5_ levels in the TRAP chambers were 10–100 times greater and ~62-fold higher than in FA chambers, respectively (78). At the end of the exposure period, rats were transported to the UCD vivarium, where they were euthanized 23 days later with 4% isoflurane (Southmedic Inc.) in medical-grade air/oxygen (2:1 v/v) mixture delivered at a rate of 1.5 L/min followed by exsanguination via perfusion of ice-cold saline as previously described (78). Rats were not euthanized by high-energy microwave irradiation, as is often recommended for lipidomic studies (79), in order to better model the human postmortem analysis. Additionally, we have previously reported that esterified oxylipins are less affected by the PMI compared with free oxylipins (42).

Prior to euthanasia, females only were anesthetized with 2%–3% isoflurane for MRI and PET imaging sessions. Males were not subjected to MRI and PET imaging. Brains from both sexes were dissected and cut in half using a stainless-steel rat brain matrix (Zivic Instruments). The entire right hemisphere was used for lipidomic measurements. Samples were immediately collected in centrifuge tubes, snap frozen in liquid nitrogen, and stored at –80°C until they were analyzed.

### MRI and PET imaging.

MRI and PET scans were performed on female rats only using scanners located at the UCD Center for Molecular and Genomic Imaging (CMGI). For all in vivo imaging procedures, the rats were anesthetized with approximately 2% isoflurane in oxygen gas and maintained on 1%–2% isoflurane. Once anesthetized, the rats were restrained in a stereotaxic apparatus and subjected to physiological monitoring (SA Instruments), and their body temperature was maintained at 37°C using warm air or a heat lamp. Respiration was maintained between 50 and 90 breaths per minute by adjusting the level of isoflurane gas percentage.

A structural T_2_-weighted MRI acquisition was obtained on a 7.0T preclinical MRI scanner (Biospec 70/30, Bruker BioSpin MRI). A 72 mm internal diameter linear transmit coil, and a 4-channel, rat-brain phased array in cross coil configuration for signal reception were used. Images were collected using the Rapid Acquisition with Repeated Echoes (RARE) pulse sequence (transaxial acquisition) over 13 minutes using the following parameters: TR/TE = 8100/60 ms; RARE factor = 8; averages = 4; field of view (FOV) = 35 × 25 mm^2^, with an in-plane data matrix of 280 × 200, resulting in a resolution of 0.125 × 0.125 mm^2^; 60 slices with a 0.5-mm thickness, spanning the entire brain.

Radiotracers for SV2A (^18^F-UCB-H) and TSPO (^18^F-PBR111) were synthesized by the CMGI based on published methods (80, 81). Anesthetized rats were administered the radiotracer, approximately 37 MBq, via tail vein catheter injection and then imaged on CMGI’s small animal PET systems (Inveon DPET or Focus 120, Siemens Healthcare). Listmode data were collected over a 60-minute period, starting 15 seconds before tail vein injection of the radiotracer. Data were reconstructed using 2 iterations of the 3-D ordered subset expectation maximization method followed by 18 iterations of a maximum a posteriori algorithm provided by the scanner vendor into 20-minute frames, and the 1,200–2,400 second frame was used for determining the SUV (82). The SUV is defined as the ratio of the activity in tissue per mL to the activity in the injected dose patient body weight (83). The ^18^F-FDG radiotracer was obtained from PETNET Solutions. The rats were anesthetized and were administered approximately 37 MBq of ^18^F-FDG via tail vein injection. The rats were kept awake during the uptake period (approximately 25 minutes), before being anesthetized and placed on the bed of the PET scanner. Thirty-minute static scans were then acquired (starting at 30 minutes after radiotracer injection).

For analysis of the images, a custom brain atlas was created (84). This atlas was aligned with the MRI of each animal using the PMOD v4.0 software tool (PMOD Technologies) to delineate the whole brain volume of interest (VOI). PET images for each rat were coregistered to its own MRI scan data via manual registration. The whole brain VOI derived from the MRI was then applied to the PET data to extract the average whole brain SUV.

### Human postmortem brain samples.

Samples were from deceased individuals enrolled in the UCD Alzheimer’s Disease Research Center (ADRC) program. During life, research participants were enrolled in IRB-approved study at UCD, and at death autopsies were performed after legal consent for autopsy was provided by appropriate family members. Prefrontal cortex from 20 individuals with no AD (control group, 10 females) and 21 individuals with AD (12 females) were obtained from the UCD ADRC brain bank (85). AD samples were diagnosed according to the National Institute on Aging (NIA) Alzheimer’s Association guidelines pathological criteria of a high likelihood of AD (86, 87). The non-AD group was defined as having low likelihood of AD based on the NIA Alzheimer’s association guidelines. The Braak NFT stage was 1.6 ± 0.5 and 5.8 ± 0.4 in the control and AD groups, respectively (88). As previously reported, the mean age of individuals without AD and with AD was 75.2 ± 5.9 and 81.3 ± 6.8 years (*P* < 0.05 by 2-tailed, unpaired *t* test) (88). The PMI was 27.6 ± 26.1 and 10.9 ± 11.2 for the non-AD control and AD groups (*P* < 0.05 by unpaired *t* test) (88). In total, 11% of controls had the apoE4 allele, where 75% of individuals with AD were apoE4^+^ (88). Other patient demographics and the presence of any copathologies are detailed in Otoki et al. (88). Fresh frozen prefrontal samples were dissected on dry ice to obtain 300–500 mg of samples for esterified (NL and PL) oxylipin analysis as described below.

### Brain lipid extraction.

Rat brain total lipids were extracted from the right hemisphere using a modified Folch method (88, 89). Brains weighing ~800 mg were weighed and transferred into 2 mL centrifuge tubes precooled and maintained on dry ice. Three zirconia beads and approximately 700 μL solution of 1 mM Na_2_EDTA and 0.9% NaCl dissolved in MilliQ water (kept at 4°C before use) were added into each centrifuge tube containing the brain samples. Because a rat brain contains ~90% water (90), the total volume of the aqueous phase was adjusted to approximately 1,420 μL (700 μL added + 720 μL coming from the brain).

Total lipids from 300 to 500 mg of human prefrontal cortex were extracted in 1,000–1,200 μL solution of 1 mM Na_2_EDTA and 0.9% KCl dissolved in MilliQ water (to make the total aqueous phase volume of ~1,500 μL after accounting for water content of the human brain).

Both rat and human brains were homogenized in a Bullet Blender (Next Advance Storm 24) for 30 seconds twice, and the resulting homogenate was transferred into 8 mL glass tubes containing 4 mL chloroform. The centrifuge tubes were then washed with 1 mL of 0.006% BHT methanol solution (precooled in a 4°C fridge before use) and vortexed for 30 seconds. The mixture in the centrifuge tubes was transferred into the 8 mL glass tubes. This step was repeated one more time to ensure that all lipids in the 2 mL centrifuge tubes were completely transferred to the 8 mL glass tubes.

The 8 mL glass tubes containing brain total lipid extracts were vortexed and centrifuged at 920*g* for 15 minutes at 0°C in a Sorvall RT 6000 centrifuge (Bio Surplus). The bottom chloroform layer from each sample extract was transferred into a new 8 mL glass tube. Chloroform (4 mL) was added to the remaining upper layer, and the samples were vortexed and centrifuged again at 920*g* for 15 minutes at 0°C. The bottom chloroform layer was transferred and combined with the first chloroform extract in the 8 mL glass tube.

The total brain lipid extract was dried under nitrogen and reconstituted in 8 mL of chloroform/isopropanol (2:1 v/v). Samples were stored in a –80°C freezer. Every 19 brain samples were accompanied by an additional method blank consisting of 800 μL of MilliQ water (instead of 800 mg of rat brain), that underwent the same extraction procedures outlined above.

### Separation of NL and PL.

Waters silica solid phase extraction (SPE) columns (Sep-Pak Silica, 1 cc, 100 mg, Waters Corporation; catalog WAT023595) were used to separate NL from polar lipids including PL (59) and any residual free oxylipins that were not removed during the Folch extraction (91). Methanol (1.5 mL) and 2:1 v/v chloroform/isopropanol (1.5 mL) were loaded onto each silica SPE column to activate and equilibrate the column. The column was loaded with 300 μL of brain total lipid extract (containing ~3 mg of total lipids) dissolved in chloroform/isopropanol (2:1 v/v), and eluted with 1.5 mL of chloroform/isopropanol (2:1 v/v). The eluent containing NL was collected in 2 mL centrifuge tubes. The column was then loaded with 1.5 mL of 95% methanol, and the eluent containing polar lipids (e.g., PL) was collected in another 2 mL centrifuge tube.

The eluent containing polar lipids in 95% methanol was adjusted to 80% methanol by adding 281 μL of MilliQ water to the 1.5 mL extract. The entire mixture was loaded onto Waters tC18 columns (Sep-Pak tC18, 1 cc, 100 mg, Waters Corporation; catalog WAT036820) prerinsed with 1 column volume of methanol and 1.5 mL of 80% methanol. The column was washed with 2 mL of 80% methanol to remove free fatty acids and free oxylipins, followed by 2 mL methanol to elute PL, which were collected in 2 mL centrifuge tubes and stored in –80°C until the hydrolysis step (below).

### Hydrolysis of NL and PL.

The collected NL and PL fractions were dried under nitrogen and dissolved in 200 μL of ice-cold extraction solvent containing 0.1 % acetic acid and 0.1% of BHT in methanol. Each sample was spiked with 10 μL of antioxidant solution containing 0.2 mg/mL BHT, EDTA, and TPP in water/methanol (1:1 v/v) and 10 μL of surrogate mix standard solution containing 2 μM of d11-11(12)-EpETrE, d11-14,15-DiHETrE, d4-6-keto-PGF1a, d4-9-HODE, d4-LTB4, d4-PGE2, d4-TXB2, d6-20-HETE, and d8-5-HETE in liquid chromatography–MS (LC-MS) grade methanol (i.e., 20 picomole per sample). Then, 200 μL of 0.25M sodium hydroxide (rat brain sample) or sodium carbonate (human brain sample) in water/methanol (1:1 v/v) was added to each sample. The mixture was vortexed and heated for 30 minutes at 60°C on a heating block to hydrolyze esterified oxylipins. After cooling for 5 minutes, 25 μL of acetic acid and 1575 μL of MilliQ water were added. The samples were vortexed and stored at –20°C (for ~1 hour) for further purification of the hydrolyzed oxylipins by SPE as described in the following section.

### Oxylipin separation by SPE.

The hydrolyzed oxylipins were isolated using Waters Oasis HLB SPE columns (3 cc, 60 mg, 30 μm particle size; Waters Corporation; catalog WAT094226) as previously described (91). The SPE columns were washed with 1 column volume of ethyl acetate and 2 column volumes of methanol, and they were preconditioned with 2 column volumes of SPE buffer containing 0.1% acetic acid and 5% methanol in MilliQ water. The hydrolyzed samples were loaded onto the columns, which were then washed with 2 column volumes of SPE buffer and dried under vacuum (approximately 15–20 psi) for 20 minutes. Oxylipins were then eluted from the columns with 0.5 mL methanol and 1.5 mL ethyl acetate, and they were collected in 2 mL centrifuge tubes. The samples were dried under nitrogen, reconstituted in 100 μL LCMS grade methanol, vortexed for 2 minutes, and centrifuged at 15,871*g* (0°C; 5424 R Centrifuge; Eppendorf AG) for 2 minutes. The samples were transferred to centrifuge filter tubes (Ultrafree-MC VV Centrifugal Filter, 0.1 μm; MilliporeSigma; catalog UFC30VV00) and centrifuged at 15,871*g* (0°C) for 20 minutes. The filtrate was transferred into 2 mL amber LC-MS vials (Phenomenex; catalog AR0-3911-13) with preslit caps (Phenomenex; catalog AR0-8972-13-B) and inserts (Waters Corporation; catalog WAT094171). Samples were stored in a –80°C freezer for ultra high-pressure LC-MS/MS (UPLC-MS/MS) analysis.

### Oxylipins analysis by UPLC-MS/MS.

A total of 76 oxylipins derived from LA, DGLA, AA, ALA, EPA, and DHA were measured with UPLC-MS/MS, using an Agilent 1290 Infinity UPLC system coupled to an Agilent 6460 Triple Quadropole mass-spectrometer (Agilent Technologies). The UPLC was equipped with an Agilent ZORBAX Eclipse Plus C18 column (2.1 × 150 mm, 1.8 μm particle size; Agilent Technologies; catalog 959759-902) to separate oxylipins. The column was kept at 45°C. The system was operated in a negative electrospray ionization mode using the optimized dynamic Multiple Reaction Monitoring (dMRM) conditions shown in Supplemental Table 1.

The temperature of the autosampler was set at 4°C, and the sample injection volume was 10 μL. Mobile phase A contained 0.1% acetic acid in MilliQ water and Mobile phase B consisted of acetonitrile/methanol (80:15 v/v) containing 0.1% acetic acid. The mobile phase gradient and pressure program were as follows: (a) 0–2 minutes, 35% B, 0.25 mL/min (this was diverted into a waste bottle and not injected into the MS); (b) 2–12 minutes, 35%–85% B, 0.25 mL/min; (c) 12–15minutes, 85% B, 0.25 mL/min; (d) 15.1–17 minutes, 85%–100% B, 0.4 mL/min; (e) 17.1–19 minutes, 100%–35% B, 0.4 mL/min; and (f) 19–20 minutes, 35% A, 0.3 mL/min. The total run time was 20 minutes.

### Statistics.

Data were analyzed on GraphPad Prism v.8.02 or SPSS 20.0 (SPSS Inc.). Data are presented as mean ± SD.

For the rat study, nondetected oxylipin values in 1, 2, or 3 samples per group were imputed by dividing the lowest observable concentration on the standard curve by the square root of 2. The number of imputed values for each group are shown in Supplemental Table 2. The effects of sex, genotype, and exposure on brain NL and PL oxylipins were compared by 3-way ANOVA. To better visualize changes per sex, a 1-way ANOVA followed by Duncan’s post hoc test was applied in male and female WT and TgF344-AD rats exposed to FA or TRAP. A 1-way ANOVA followed by Duncan’s post hoc test was also used to examine the effects of genotype and exposure on PET imaging markers (TSPO, SV2A, and glucose metabolism) within female rats. In an exploratory manner, we tested for associations between esterified oxylipins and PET brain markers of AD pathology using Pearson’s correlation.

In the postmortem human study, oxylipins with less than 30% missing values were imputed by dividing the lowest observable concentration on the standard curve by the square root of 2. Data were analyzed using multiple linear regression analysis. Log-transformed concentrations of each oxylipin was set as the dependent variable, and group, sex, mean-centered PMI and mean centered age were tested for main effects. Interaction effects were tested between group and age as well as between group and sex. Linear plots and Pearson’s correlation coefficient (*r* value) were used to visualize significant associations. Statistical significance was accepted at *P* < 0.05.

### Study approval.

Animal experiments were conducted according to the *Guide for the Care and Use of Laboratory Animals* (National Academies Press, 2011) and were approved by the UCD IACUC (protocol no. 22337). For the postmortem human study, the IRB of UCD approved the study (protocol no. 215830), and written consent was obtained for each participant for autopsy evaluations. Autopsy brain tissue was free of personal identifiers (as determined by the Health Insurance Portability and Accountability Act [HIPAA]), and proper institutional, state, and federal guidelines were followed related to human postmortem tissue research.

### Data availability.

Raw data corresponding to the animal and human studies are provided in the appended excel sheet supplement.

## Author contributions

QS contributed in conducting experiments, acquiring data, analyzing data, and writing initial manuscript draft. YO contributed in conducting experiments and acquiring data. KTP contributed in conducting experiments. NL contributed in analyzing data. AEV contributed in conducting experiments and acquiring data. CDW contributed in conducting experiments. DJR contributed in conducting experiments, acquiring data, analyzing data, and study design. AJC contributed in conducting experiments, acquiring data, analyzing data, and study design. KJB contributed in conducting experiments, acquiring data, providing reagents, and study design. ASW contributed to study design. LWJ contributed in conducting experiments, acquiring data, analyzing data, and study design. BND contributed in conducting experiments, acquiring data, analyzing data, and study design. DJH contributed in acquiring data, analyzing data, and study design. PJL contributed in analyzing data and study design. AYT contributed in analyzing data and study design.

## Supplementary Material

Supplemental data

Supporting data values

## Figures and Tables

**Table 1:**
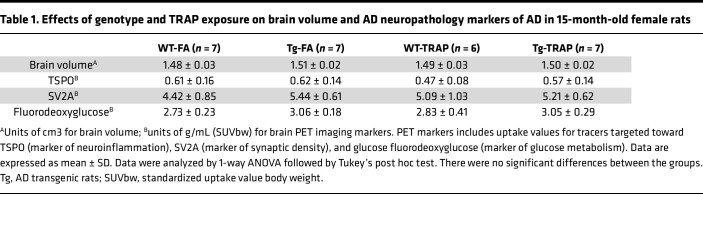
Effects of genotype and TRAP exposure on brain volume and AD neuropathology markers of AD in 15-month-old female rats

**Table 2:**
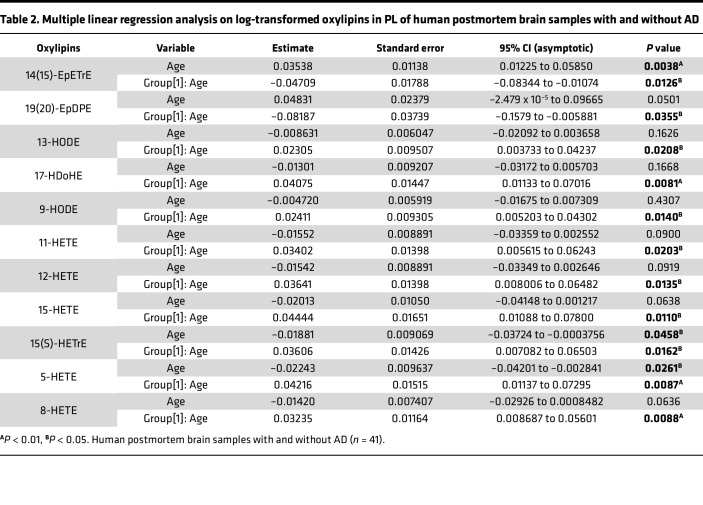
Multiple linear regression analysis on log-transformed oxylipins in PL of human postmortem brain samples with and without AD

## Version 1 05/13/2025

In-Press Preview

## Version 2 08/08/2025

Electronic publication
