# Supplementary material for: Air pollution and Alzheimer disease phenotype deplete esterified proresolving lipid mediator reserves in the brain
Source: JCI Insight. 2025 May 13;10(15):e175917. doi: 10.1172/jci.insight.175917 (PMC12348674; doi:10.1172/jci.insight.175917)
Supplement: Supplemental data [file jciinsight-10-175917-s008.pdf]

Supplementary information

Air pollution and Alzheimer disease phenotype deplete esterified pro-resolving lipid mediator reserves in the brain

Ameer Y. Taha[1,2,3], Qing Shen[1], Yurika Otoki[1.4], Nuanyi Liang[1], Kelley T. Patten[5], Anthony E. Valenzuela[5], Christopher Wallis[6], Douglas J Rowland[7], Abhijit J. Chaudhari[7,8], Keith J. Bein[9,10], Anthony S. Wexler[6,10], Lee-Way Jin[11], Brittany N. Dugger[11], Danielle J. Harvey[12], Pamela J. Lein[5,13]

[1]Department of Food Science and Technology, College of Agriculture and Environmental Sciences, University of California, Davis, CA, USA

[2]Center for Neuroscience, University of California, Davis, One Shields Avenue, Davis, CA 95616, USA.

[3]West Coast Metabolomics Center, Genome Center, University of California, Davis, CA, USA.

[4]Food and Biodynamic Laboratory, Graduate School of Agricultural Science, Tohoku University, Sendai, Miyagi, Japan.

[5]Department of Molecular Biosciences, School of Veterinary Medicine, University of California, Davis, CA, USA

[6]Air Quality Research Center, University of California, Davis, CA, USA

[7]Center for Genomic and Molecular Imaging, University of California, Davis, CA, USA

[8]Department of Radiology, School of Medicine, University of California, Davis, Sacramento, CA, USA

[9]Center for Health and the Environment, University of California, Davis, CA, USA

[10]Departments of Mechanical and Aerospace Engineering, Civil and Environmental Engineering, and Land, Air and Water Resources, University of California, Davis, CA, USA

[11]Department of Pathology and Laboratory Medicine, University of California - Davis School of Medicine, Sacramento, CA, USA.

[12]Department of Public Health Sciences, University of California - Davis, Davis, CA, USA

[13]The MIND Institute, School of Medicine, University of California, Davis, Sacramento, CA, USA

* Corresponding author: Ameer Y. Taha

Department of Food Science and Technology, College of Agriculture and Environmental Sciences, One Shields Avenue, University of California, Davis, CA, USA

Phone: +1 530 752 7096; E-mail: ataha@ucdavis.edu

Supplementary Figure 1: Pathway of main oxylipins that were quantified in the present study.

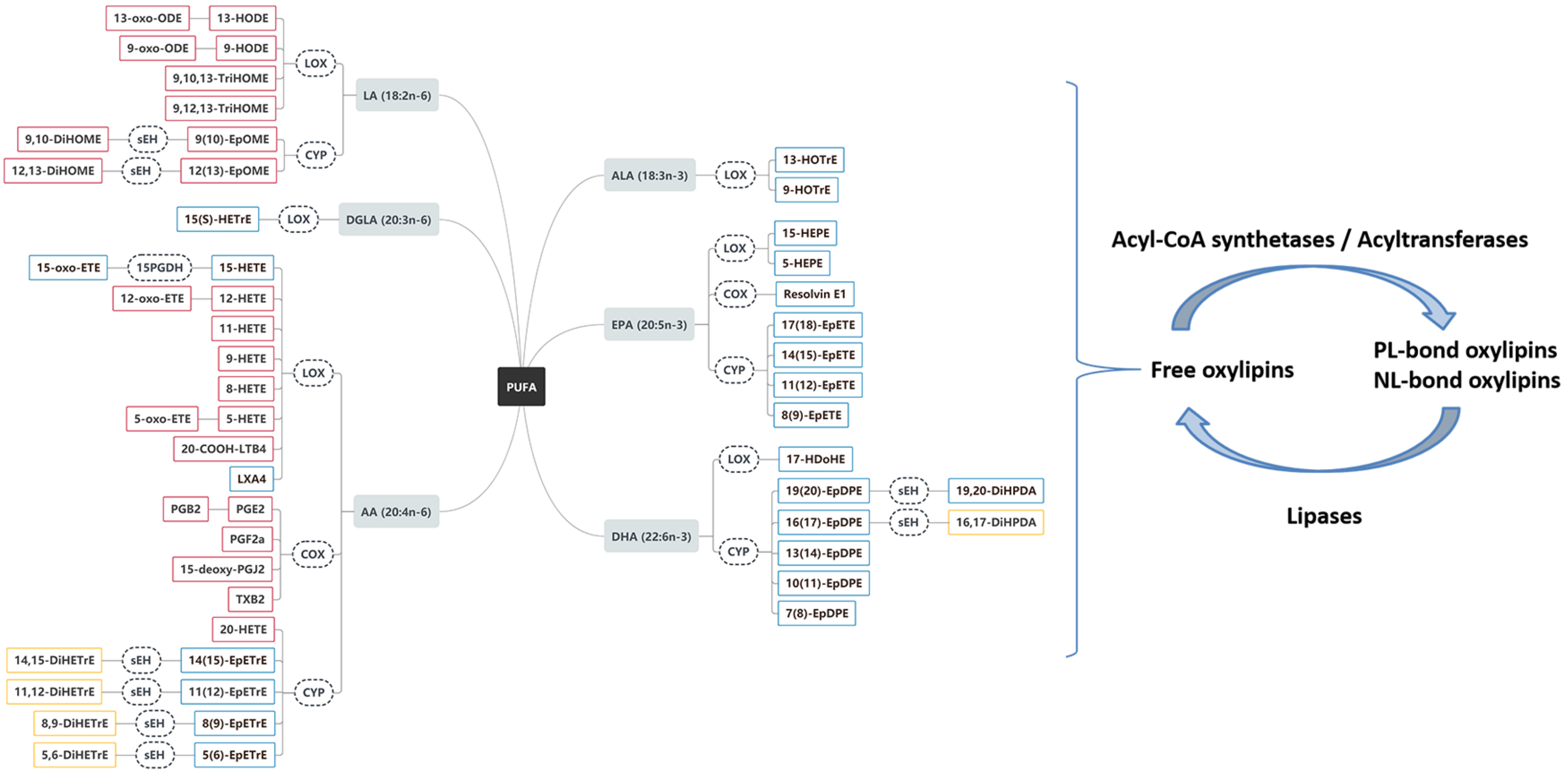

Frames surrounding each oxylipin classify whether it is pro- or anti-inflammatory. Oxylipins with red frames have pro-inflammatory effects; oxylipins with blue frames have anti-inflammatory / pro-resolving effects; and) oxylipins with yellow frames do not have well-characterized pro-or anti-inflammatory effects. Esterified oxylipins within phospholipids (PL) or neutral lipids (NL) pool can be released via lipase enzymes to generate free (unesterified) oxylipins. Free oxylipins can also be sequestered into NL and PL pools via acyl-CoA synthetase and acyltransferase enzymes. Both lipase-mediated release and acyl-CoA synthetase/acyltraferase-mediated

sequestration regulate the availability of free oxylipins. Abbreviations: PUFA, polyunsaturated fatty acid; LA, linoleic acid; DGLA, dihomo-gamma-linoleic acid; AA, arachidonic acid; ALA, alpha-linolenic acid; EPA, eicosapentaenoic acid; DHA, docosahexaenoic acid; COX, cyclooxygenase; CYP, cytochrome P450; LOX, lipoxygenase; sEH, soluble epoxide hydrolase; 15-PGDH, 15-hydroxyprostaglandin dehydrogenase; DiHETE, dihydroxyeicosatetraenoic acid; DiHETrE, dihydroxyeicosatrienoic acid; DiHOME, dihydroxyoctadecenoic acid; DiHDPA, dihydroxydocosapentaenoic acid; EpDPE, epoxydocosapentaenoic acid; EpETE, epoxyeicosatetraenoic acid; EpETrE, epoxyeicosatrienoic acid; EpOME, epoxyoctadecenoic acid; HDoHE, hydroxydocosahexaenoic acid; HEPE, hydroxyeicosapentaenoic acid; HETE, hydroxyeicosatetraenoic acid; HETrE, hydroxyeicosatrienoic acid; HODE, hydroxyoctadecadienoic acid; HOTrE, hydroxyoctadecatrienoic acid; oxo-ETE, oxo-eicosatetraenoic acid; oxo-ODE, oxo-octadecadienoic acid; TriHOME, trihydroxyoctadecenoic acid; LX, lipoxin; PG, prostaglandin; LT, leukotriene.

Supplementary Table 1. Retention time, parent ion, product ion, and internal standards used in neutral lipids (NLs) and phospholipids (PLs) of the 76 quantified oxylipins in rat brain samples.

Oxylipins	Compound name	Internal standards		RT (min)	Precursor Ion (m/z)	Product Ion (m/z)
		NL fraction	PL fraction			
**Surrogates**						
d11-11(12)-EpETrE	d11-11(12)-epoxyeicosatrienoic acid	NA	NA	13.72928	330.2	167.2
d11-14,15-DiHETrE	d11-14,15-dihydroxyeicosatrienoic acid	NA	NA	10.11682	348.2	207.1
d4-6-keto-PGF1a	d4-6-keto-Prostaglandin F1 alpha	NA	NA	3.7867	373.3	167.1
d4-9-HODE	d4-9-hydroxyoctadecadienoic acid	NA	NA	11.90957	299.2	172.3
d4-LTB4	d4-Leukotriene B4	NA	NA	9.104717	339.2	197.2
d4-PGE2	d4-Prostaglandin E2	NA	NA	5.776367	355.2	275.3
d4-TXB2	d4-Tromboxane B2	NA	NA	4.87205	373.3	173.2
d6-20-HETE	d6-20-hydroxyeicosatetraenoic acid	NA	NA	11.27772	325.2	281.2
d8-5-HETE	d8-5-hydroxyeicosatetraenoic acid	NA	NA	12.90778	327.2	116.1
**LA-derived oxylipins**						
13-HODE	13-hydroxyoctadecadienoic acid	d4-9HODE	d4-9HODE	11.91842	295.2	195.2
9-HODE	9-hydroxyoctadecadienoic acid	d4-9HODE	d4-9HODE	11.98537	295.2	171.1
13-oxo-ODE	13-oxo-octadecadienoic acid	d4-9HODE	d4-9HODE	12.34552	293.2	195.1
9-oxo-ODE	9-oxo-octadecadienoic acid	d4-9HODE	d4-9HODE	12.5964	293.2	185.1
12(13)-EpOME	12(13)-epoxyoctadecamonoenoic acid	d-11-11(12)EpEtrE	d8-5-HETE	13.41593	295.3	195.2
9(10)-EpOME	9(10)-epoxyoctadecamonoenoic acid	d-11-11(12)EpEtrE	d8-5-HETE	13.5412	295.3	171.2
12,13-DiHOME	12,13-dihydroxyoctadecamonoenoic acid	d11-14,15-DiHETrE	d11-14,15-DiHETrE	9.579483	313.2	183.2
9,10-DiHOME	9,10-dihydroxyoctadecamonoenoic acid	d11-14,15-DiHETrE	d11-14,15-DiHETrE	9.854117	313.2	201.2
9,10,13-TriHOME	9,10,13-trihydroxyoctadecamonoenoic acid	d4-LTB4	d4-LTB4	5.53055	329.2	171.1
9,12,13-TriHOME	9,12,13-trihydroxyoctadecamonoenoic acid	d4-LTB4	d4-LTB4	5.421833	329.2	211.1
**DGLA-derived oxylipins**						
15(S)-HETrE	15(S)-hydroxyeicosatrienoic acid	d8-5-HETE	d8-5-HETE	12.84102	321.2	221.2
LTB3	Leukotriene B3	d11-14,15-DiHETrE	d11-14,15-DiHETrE	10.2935	337.2	195.2
PGD1	Prostaglandin D1	d4-LTB4	d4-LTB4	6.12885	353.3	317.2
PGE1	Prostaglandin E1	d4-LTB4	d4-LTB4	5.985983	353.3	317.2
**AA-derived oxylipins**						
20-HETE	20-hydroxyeicosatetraenoic acid	d6-20-HETE	d6-20-HETE	11.32837	319.2	275.1

15-HETE	15-hydroxyeicosatetraenoic acid	d4-9HODE	d4-9HODE	12.20508	319.2	219.2
12-HETE	12-hydroxyeicosatetraenoic acid	d8-5-HETE	d8-5-HETE	12.65718	319.2	179.2
11-HETE	11-hydroxyeicosatetraenoic acid	d4-9HODE	d4-9HODE	12.4396	319.2	167.2
9-HETE	9-hydroxyeicosatetraenoic acid	d8-5-HETE	d8-5-HETE	12.8331	319.2	167.2
8-HETE	8-hydroxyeicosatetraenoic acid	d8-5-HETE	d8-5-HETE	12.6403	319.2	155.2
5-HETE	5-hydroxyeicosatetraenoic acid	d8-5-HETE	d8-5-HETE	12.984	319.2	115.1
15-oxo-ETE	15-oxo-eicosatetraenoic acid	d4-9HODE	d4-9HODE	12.55912	317.2	113.1
12-oxo-ETE	12-oxo-eicosatetraenoic acid	d8-5-HETE	d8-5-HETE	12.92008	317.2	153.1
5-oxo-ETE	5-oxo-eicosatetraenoic acid	d-11-11(12)EpEtrE	d8-5-HETE	13.69905	317.2	273.2
8,15-DiHETE	8,15-dihydroxyeicosatetraenoic acid	d11-14,15-DiHETrE	d11-14,15-DiHETrE	8.581667	335.2	235.2
5,15-DiHETE	5,15-dihydroxyeicosatetraenoic acid	d11-14,15-DiHETrE	d11-14,15-DiHETrE	8.84965	335.2	173.2
14(15)-EpETrE	14(15)-epoxyeicosatrienoic acid	d-11-11(12)EpEtrE	d8-5-HETE	13.45405	319.2	219.3
11(12)-EpETrE	11(12)-epoxyeicosatrienoic acid	d-11-11(12)EpEtrE	d8-5-HETE	13.8051	319.2	167.2
8(9)-EpETrE	8(9)-epoxyeicosatrienoic acid	d-11-11(12)EpEtrE	d8-5-HETE	13.93835	319.2	167.2
5(6)-EpETrE	5(6)-epoxyeicosatrienoic acid	d-11-11(12)EpEtrE	d8-5-HETE	14.08898	319.2	191.1
14,15-DiHETrE	14,15-dihydroxyeicosatrienoic acid	d11-14,15-DiHETrE	d11-14,15-DiHETrE	10.2011	337.2	207.1
11,12-DiHETrE	11,12-dihydroxyeicosatrienoic acid	d11-14,15-DiHETrE	d11-14,15-DiHETrE	10.61052	337.2	167.1
8,9-DiHETrE	8,9-dihydroxyeicosatrienoic acid	d11-14,15-DiHETrE	d11-14,15-DiHETrE	10.95115	337.2	127.1
5,6-DiHETrE	5,6-dihydroxyeicosatrienoic acid	d6-20-HETE	d6-20-HETE	11.4268	337.2	145.1
20-COOH-LTB4	20-COOH- Leukotriene B4	d4-LTB4	d4-LTB4	3.774633	365.2	347.2
15-deoxy-PGJ2	15-deoxy-Prostaglandin J2	d6-20-HETE	d6-20-HETE	11.22373	315.2	271.2
20-OH-LTB4	20-OH-Leukotriene B4	d4-LTB4	d4-LTB4	4.034333	351.2	195.2
6-keto-PGF1a	6-keto-prostaglandin F1 alpha	d4-LTB4	d4-LTB4	3.888733	369.3	163.2
6-trans-LTB4	6-trans-leukotriene B4	d4-LTB4	d4-LTB4	8.841317	335.2	195.1
LTB4	Leukotriene B4	d4-LTB4	d4-LTB4	9.141617	335.2	195.1
LTC4	Leukotriene C4	d4-LTB4	d4-LTB4	7.51035	624.3	272.1
LTD4	Leukotriene D4	d4-LTB4	d4-LTB4	6.342133	495.3	177.1
LTE4	Leukotriene E4	d4-LTB4	d4-LTB4	7.604183	438.2	333.1
LXA4	Lipoxin A4	d4-LTB4	d4-LTB4	6.655133	351.2	115.2
PGB2	Prostaglandin B2	d4-LTB4	d4-LTB4	8.025267	333.3	175.1
PGD2	Prostaglandin D2	d4-LTB4	d4-LTB4	6.164517	351.2	271.3
PGE2	Prostaglandin E2	d4-LTB4	d4-LTB4	5.80365	351.2	271.3
PGF2a	Prostaglandin F2 alpha	d4-LTB4	d4-LTB4	5.535717	353.2	309.2
PGJ2	Prostaglandin J2	d4-LTB4	d4-LTB4	7.924717	333.3	189.2
TXB2	Tromboxane B2	d4-LTB4	d4-LTB4	4.891267	369.2	169.1

**ALA-derived oxylipins**						
13-HOTrE	13- hydroxyoctadecatrienoic acid	d6-20-HETE	d6-20-HETE	10.95783	293.2	195.1
9-HOTrE	9- hydroxyoctadecatrienoic acid	d11-14,15-DiHETrE	d11-14,15-DiHETrE	10.774	293.2	171.2
**EPA-derived oxylipins**						
15-HEPE	15-hydroxyeicosapentaenoic acid	d6-20-HETE	d6-20-HETE	11.29557	317.2	219.2
12-HEPE	12-hydroxyeicosapentaenoic acid	d6-20-HETE	d6-20-HETE	11.53805	317.2	179.2
8-HEPE	8-hydroxyeicosapentaenoic acid	d6-20-HETE	d6-20-HETE	11.46287	317.2	155.2
5-HEPE	5-hydroxyeicosapentaenoic acid	d4-9HODE	d4-9HODE	11.7975	317.2	115.1
17(18)-EpETE	17(18)-epoxyeicosateteaenoic acid	d4-9HODE	d4-9HODE	12.3076	317.2	215.2
14(15)-EpETE	14(15)-epoxyeicosatetraenoic acid	d4-9HODE	d4-9HODE	12.5928	317.2	207.2
11(12)-EpETE	11(12)-epoxyeicosateteaenoic acid	d4-9HODE	d4-9HODE	12.7612	317.2	167.2
8(9)-EpETE	8(9)-epoxyeicosateteaenoic acid	d8-5-HETE	d8-5-HETE	12.76955	317.2	127.2
17,18-DiHETE	17,18-dihydroxyeicosatetraenoic acid	d11-14,15-DiHETrE	d11-14,15-DiHETrE	8.973383	335.3	247.2
14,15-DiHETE	14,15-dihydroxyeicosatetraenoic acid	d11-14,15-DiHETrE	d11-14,15-DiHETrE	9.324117	335.3	207.2
11,12-DiHETE	11,12-dihydroxy-5Z,8Z,14Z,17Z-eicosatetraenoic acid	d11-14,15-DiHETrE	d11-14,15-DiHETrE	9.44115	335.5	167.0
8,9-DiHETE	8,9-dihydroxy-5Z,11Z,14Z,17Z-eicosatetraenoic acid	d11-14,15-DiHETrE	d11-14,15-DiHETrE	9.6757	335.5	126.9
5,6-DiHETE	5,6-dihydroxyeicosatetraenoic acid	d11-14,15-DiHETrE	d11-14,15-DiHETrE	8.84965	335.2	115.2
PGD3	Prostaglandin D3	d4-LTB4	d4-LTB4	5.261533	349.3	269.2
PGE3	Prostaglandin E3	d4-LTB4	d4-LTB4	4.9695	349.3	269.2
Resolvin E1	Resolvin E1	d4-LTB4	d4-LTB4	3.868317	349.3	195.0
**DHA-derived oxylipins**						
17-HDoHE	17- hydroxydocosahexaenoic acid	d4-9HODE	d4-9HODE	12.26277	343.2	281.2
19(20)-EpDPE	19(20)-epoxydocosapentaenoic acid	d-11-11(12)EpEtrE	d8-5-HETE	13.26633	343.2	241.2
16(17)-EpDPE	16(17)-epoxydocosapentaenoic acid	d-11-11(12)EpEtrE	d8-5-HETE	13.5339	343.2	233.2
13(14)-EpDPE	13(14)-epoxydocosapentaenoic acid	d-11-11(12)EpEtrE	d8-5-HETE	13.58403	343.2	193.2
10(11)-EpDPE	10(11)-epoxydocosapentaenoic acid	d-11-11(12)EpEtrE	d8-5-HETE	13.65088	343.2	153.2
7(8)-EpDPE	7(8)-epoxydocosapentaenoic acid	d-11-11(12)EpEtrE	d8-5-HETE	13.80957	343.2	113.1
19,20-DiHDPA	19,20-dihydroxydocosapentaenoic acid	d11-14,15-DiHETrE	d11-14,15-DiHETrE	10.18212	361.5	273.1
16,17-DiHDPA	16,17-dihydroxydocosapentaenoic acid	d11-14,15-DiHETrE	d11-14,15-DiHETrE	10.49112	361.5	233.1

NA: not applicable.

Low response of d-11-11(12)-EpETrE in many phospholipids fraction samples.

Supplementary Table 2. Number of imputed oxylipin values in each group for compounds that were missing 1, 2, or 3 values.

**Group**	F-WT-FA	F-Tg-FA	F- WT-TRAP	F- Tg-TRAP	M- WT-FA	M-Tg-FA	M-WT-TRAP	M-Tg-TRAP
**N**	7	7	6	7	7	7	7	6
**Fraction**					**Neutral lipids**			
20-COOH-LTB4	1	0	0	1	2	0	0	1
PGF2a	0	0	0	0	1	0	0	0
PGE2	NA	NA	NA	NA	2	0	2	1
PGB2	1	0	1	2	3	1	1	1
8,9-DiHETrE	0	0	0	0	0	1	0	0
15-deoxy-PGJ2	0	0	0	0	0	0	0	1
17(18)-EpETE	0	2	2	0	NA	NA	NA	NA
13-oxo-ODE	0	1	0	1	1	2	1	0
14(15)-EpETE	0	2	1	0	1	1	1	1
8(9)-EpETE	0	0	1	0	0	0	0	0
12-oxo-ETE	1	1	0	2	1	2	0	2
**Fraction**					**Phospholipids**			
20-COOH-LTB4	0	1	0	1	0	2	1	0
PGE2	1	3	1	3	NA	NA	NA	NA
8,9-DiHETrE	0	2	0	1	1	1	2	1
15-deoxy-PGJ2	1	0	1	1	2	0	0	0
17(18)-EpETE	1	2	1	1	1	0	1	1
14(15)-EpETE	NA	NA	NA	NA	2	1	2	1
8(9)-EpETE	0	0	0	0	1	0	0	0
10(11)-EpDPE	0	2	0	1	0	1	0	0
11(12)-EpETrE	2	1	2	1	NA	NA	NA	NA
7(8)-EpDPE	3	3	2	3	NA	NA	NA	NA

NA: not applicable (more than 3 values are missing in one group).

Data are expressed as mean$^{n=\text{detected number}}$(n<3) or mean±SD $^{n=\text{detected number}}$(n≥3)

Supplementary Table 3. Three-way ANOVA *p* value results of brain oxylipins in neutral lipids (NLs) of 15-month old rats (n=54)

**Oxylipins**	**Source of variation**						
	**Sex**	**Exposure**	**Genotype**	**Sex × Exposure**	**Sex × Genotype**	**Exposure × Genotype**	**Sex × Exposure × Genotype**
**LA-derived oxylipins**							
13-HODE	ns	ns	ns	ns	ns	ns	ns
9-HODE	ns	ns	ns	ns	ns	ns	ns
13-oxo-ODE	ns	ns	ns	ns	ns	ns	ns
9-oxo-ODE	ns	ns	ns	ns	ns	ns	ns
12(13)-EpOME	ns	ns	ns	ns	ns	ns	ns
9(10)-EpOME	ns	ns	ns	ns	ns	ns	ns
12,13-DiHOME	ns	ns	ns	ns	ns	ns	ns
9,10-DiHOME	ns	ns	ns	ns	ns	ns	ns
9,12,13-TriHOME	ns	ns	ns	ns	ns	ns	ns
9,10,13-TriHOME	ns	ns	ns	ns	ns	ns	ns
**DGLA-derived oxylipins**							
15(S)-HETrE	0.0061	ns	0.0096	ns	ns	ns	ns
**AA-derived oxylipins**							
20-HETE	ns	ns	ns	ns	ns	ns	ns
15-HETE	ns	ns	0.0363	ns	ns	ns	ns
12-HETE	ns	ns	ns	ns	ns	ns	0.0265
11-HETE	ns	ns	0.0162	ns	ns	ns	ns
9-HETE	ns	ns	ns	ns	ns	ns	0.0059
8-HETE	ns	ns	ns	ns	ns	ns	0.0158
5-HETE	ns	ns	ns	ns	ns	ns	ns
15-oxo-ETE	ns	ns	ns	ns	ns	ns	ns
12-oxo-ETE	0.0219	ns	ns	0.0197	ns	ns	ns
5-oxo-ETE	ns	ns	ns	ns	ns	ns	ns
14(15)-EpETrE	ns	ns	ns	ns	ns	ns	ns
11(12)-EpETrE	ns	ns	0.0271	ns	ns	ns	ns
8(9)-EpETrE	ns	ns	ns	ns	ns	ns	ns
5(6)-EpETrE	0.0447	ns	ns	ns	ns	ns	ns
14,15-DiHETrE	ns	ns	0.0002	ns	ns	ns	ns

11,12-DiHETrE	ns	ns	0.0077	ns	ns	ns	ns
8,9-DiHETrE	ns	ns	0.0123	ns	ns	ns	ns
5,6-DiHETrE	ns	ns	ns	ns	ns	ns	ns
20-COOH-LTB4	ns	ns	ns	ns	ns	ns	ns
15-deoxy-PGJ2	ns	ns	ns	0.0235	ns	ns	ns
LXA4	0.0020	ns	ns	ns	ns	ns	ns
PGB2	ns	ns	ns	ns	ns	ns	ns
PGF2a	ns	ns	ns	ns	ns	ns	ns
**ALA-derived oxylipins**							
13-HOTrE	ns	ns	ns	ns	ns	ns	0.0457
9-HOTrE	ns	ns	ns	ns	ns	ns	ns
**EPA-derived oxylipins**							
15-HEPE	ns	ns	ns	ns	ns	ns	ns
5-HEPE	ns	ns	ns	ns	ns	ns	ns
14(15)-EpETE	ns	ns	ns	ns	ns	ns	ns
11(12)-EpETE	0.0057	ns	0.0020	ns	ns	ns	ns
8(9)-EpETE	ns	ns	ns	ns	ns	ns	ns
Resolvin E1	0.0091	ns	ns	ns	ns	ns	ns
**DHA-derived oxylipins**							
17-HDoHE	ns	ns	ns	ns	ns	ns	ns
19(20)-EpDPE	0.0102	ns	0.0394	ns	ns	ns	ns
16(17)-EpDPE	0.0164	ns	ns	ns	ns	ns	ns
13(14)-EpDPE	0.0192	ns	ns	ns	ns	ns	ns
10(11)-EpDPE	0.0178	ns	ns	ns	ns	ns	ns
7(8)-EpDPE	0.0086	ns	ns	ns	ns	ns	ns
19,20-DiHDPA	ns	ns	0.0151	ns	0.0467	ns	ns
16,17-DiHDPA	0.0023	ns	0.0242	ns	ns	ns	ns

ns: not significant, $p \geq 0.05$

Main sex effects were detected for DGLA-derived 15(S)-HETrE, AA-derived 12-oxo-ETE, 5(6)-EpETrE, and LXA4, EPA-derived 11(12)-EpETE and Resolvin E1, and most DHA-derived oxylipins, i.e., 19(20)-EpDPE, 16(17)-EpDPE, 13(14)-EpDPE, 10(11)-EpDPE, 7(8)-EpDPE, and 16,17-DiHDPA, which were all higher by 16% to 65% in brain NLs of females compared to males. Genotype effects were significant for DGLA-derived 15(S)-HETrE, AA-derived 15-HETE, 11-HETE, 11(12)-EpETrE, 14,15-DiHETrE, 11,12-DiHETrE, and 8,9-DiHETrE, EPA-derived 11(12)-EpETE, and DHA-derived 19(20)-EpDPE, 19,20-DiHDPA and 16,17-DiHDPA NLs ($p < 0.05$). There were no significant main effects of TRAP exposure on NL-bound oxylipins.

A significant sex and exposure interaction was observed for AA-derived 12-oxo-ETE and 15-deoxy-PGJ2. Sex and genotype interaction was significant for DHA-derived 19,20-DiHDPA ($p < 0.05$). A significant 3-way sex, genotype and TRAP interaction was detected for AA-derived 12-HETE, 9-HETE and 8-HETE, and ALA-derived 13-HOTrE ($p < 0.05$).

Supplementary Table 4. Oxylipin concentrations in brain neutral lipids (NLs) of 15-month old rats (n=54). A one-way ANOVA followed by Duncan's post-hoc test was applied per sex, to test for significant differences between wildtype (WT) and transgenic (Tg) rats exposed to filtered air (FA) or traffic-related air pollution (TRAP). Data are expressed as mean ± SD. WT: wildtype rats; Tg: Alzheimer's Disease transgenic rats; TRAP: traffic-related air pollution; FA: filtered air.

Oxylipins (pmol/g)	Female (n=27)				Male (n=27)				Blank (n=3)
	WT-FA (n=7)	Tg-FA (n=7)	WT-TRAP (n=6)	Tg-TRAP (n=7)	WT-FA (n=7)	Tg-FA (n=7)	WT-TRAP (n=7)	Tg-TRAP (n=6)	
**LA-derived oxylipins**									
13-HODE	219.1±249.1	98.49±81.53	160.4±259.88	271.85±366.01	92.82±83.56	79.35±48.9	134.74±111.41	192±233.02	115.28±77.73
9-HODE	51.31±46.89	25.76±18.96	44.93±69.11	65.2±70.75	21.75±13.96	21.61±12.41	36.86±32.03	45.22±51.66	28.89±23.47
13-oxo-ODE	26.39±39.33	13.49±15.56	17.28±30.38	40.32±59.76	11.57±14.84	8.1±7.87	14.3±18.95	24.2±33.54	38.07$^{n=1}$
9-oxo-ODE	11.97±14.59	4.46±4.07	7.77±11.24	13.03±12.48	3.48±1.46	4.28±2.49	7.1±8.13	6.92±6.76	12.51±10.3
12(13)-EpOME	88.42±60.28	66.23±36.9	82.14±75.28	84.01±43.44	77.89±79.83	59.15±17.18	89.66±67.52	115.85±98.14	69.97±85.05
9(10)-EpOME	45.58±32.65	32.36±20.73	37.91±33.96	43.05±31.76	42.53±54.22	28.62±8.82	40.82±27.47	63.98±63.48	33.49±40.53
12,13-DiHOME	3.64±1.83	3.22±1.1	4.06±2.31	5.04±3.53	5.44±4.35	3.95±1.95	4.21±2.36	7.25±8.54	3.41±2.12
9,10-DiHOME	4.01±1.48	3.24±1.75	3.9±3.03	5.86±4.83	5.93±6.02	3.96±2.22	4.5±2.2	9.04±11.47	4.23±3.23
9,12,13-TriHOME	139.12±150.24	72.33±46.1	80.9±85.32	304.55±614.49	250.38±438.66	64.57±40.12	108.28±68.92	266.89±373.63	90.98±90.46
9,10,13-TriHOME	34.85±39.13	18.26±10.47	22.62±22.66	78.11±154.01	67.54±117.78	17.96±15.4	26.91±19.17	78.16±106.07	21.2±21.39
**DGLA-derived oxylipins**									
15(S)-HETrE	4.17±0.71^{a}	2.53±0.92^{b}	3.89±0.68^{a}	3.38±0.63^{a}	2.66±0.4	2.75±1.38	3.16±1.23	2.61±0.65	0.18$^{n=2}$
LTB3	ND	ND	ND	ND	ND	ND	ND	ND	ND
PGD1	ND	ND	ND	ND	ND	ND	ND	ND	ND
PGE1	ND	ND	ND	ND	ND	ND	ND	ND	ND
**AA-derived oxylipins**									
20-HETE	175.36±18.52^{a}	127.19±41.26^{b}	154.49±43.45^{ab}	137.35±46.22^{ab}	149.18±28.27	132.74±36.66	140.21±26.69	164.69±23.7	124.21$^{n=2}$
15-HETE	30.13±3.17^{a}	23.60±6.07^{b}	31.96±4.69^{a}	30.01±4.95^{a}	25.88±4.07	25.3±11.27	28.27±6.05	23.04±3.94	3.19±5.14
12-HETE	9.79±6.4	6.21±1.88	7.46±2.48	7.25±3.07	8.46±4.11	12.14±9.38	10.54±4.02	5.44±1.07	1.59$^{n=2}$
11-HETE	25.25±2.33^{a}	18.70±5.70^{b}	26.33±5.87^{a}	21.67±3.94^{ab}	21.43±2.51	21.79±8.74	23.02±4.35	19.85±4.75	2.15±3.25
9-HETE	7.22±1.75^{ab}	6.39±1.43^{ab}	4.99±2.93^{a}	7.86±2.11^{b}	5.45±2.17^{ab}	7.51±2.16^{a}	5.76±3.37^{ab}	4.48±0.98^{b}	0.81$^{n=1}$
8-HETE	5.45±1.29	4.16±1.5	4.62±1.38	4.77±1.98	4.01±1.36^{ab}	4.45±2.16^{ab}	5.31±1.37^{a}	2.93±1.02^{b}	0.27$^{n=1}$
5-HETE	11.75±2.38	10.17±4.62	10.75±2.12	12.56±3.66	9.72±3.17	9.62±6	11.97±4.15	8.39±1.84	2.98±4.43
15-oxo-ETE	2.31±1.03	2±0.46	2.72±0.99	2.22±0.51	2.02±0.61	2.52±0.88	1.95±0.69	1.71±0.45	3.34±5.7
12-oxo-ETE	0.82±0.40^{a}	0.83±0.21^{a}	1.79±1.25^{b}	0.93±0.64^{a}	0.98±0.54	0.68±0.51	0.64±0.28	0.55±0.3	ND

5-oxo-ETE	21.1±8.25[ab]	14.46±6.51[a]	25.96±7.32[b]	16.04±6.56[a]	19.56±3.4	19.86±8.62	45.52±68.57	14.99±8.48	2.84$^{n=1}$
8,15-DiHETE	ND	ND	ND	ND	ND	ND	ND	ND	ND
5,15-DiHETE	ND	ND	ND	ND	ND	ND	ND	ND	ND
14(15)-EpETrE	526.87±83.14	395.06±115.73	515.71±196.71	388.71±127.99	393.82±80.33	408.88±227.19	419.53±152.29	357.85±110.83	14.72±21.35
11(12)-EpETrE	221.34±32.65[a]	150.68±47.28[b]	189.67±48.55[ab]	162.31±31.12[b]	153.2±31.69	161.4±84.14	173.08±48.75	143.22±34.76	1.27$^{n=1}$
8(9)-EpETrE	136.16±17	106.31±25.77	133.5±27.21	115.18±27.33	108.23±25.29	119.7±42.6	116.64±26.64	103.49±20.18	29.48$^{n=2}$
5(6)-EpETrE	201.73±32.35	165.84±43.29	194.79±32.14	171.4±45.74	148.67±50.62	178.76±70.56	162.52±35.05	140.43±42.75	7.03±11.48
14,15-DiHETrE	3.02±0.45[a]	1.72±0.72[b]	2.39±0.77[ab]	1.71±0.51[b]	1.92±0.6	1.69±0.86	2.29±0.48	1.66±0.54	0.61$^{n=1}$
11,12-DiHETrE	2.14±0.42[a]	1.50±0.82[ab]	1.97±0.78[ab]	1.33±0.35[b]	1.62±0.57	1.38±0.88	1.64±0.57	1.26±0.4	0.19$^{n=2}$
8,9-DiHETrE	2.6±0.7	1.79±1.35	2.56±1.16	1.65±0.94	1.8±0.59	1.51±0.9	2.04±0.84	1.42±0.6	0.28$^{n=1}$
5,6-DiHETrE	8.18±6.92	6.83±5.3	5.37±0.6	4.64±1	4.84±1.37	7.22±5.12	5.37±1.32	4.8±1.21	0.16$^{n=1}$
20-COOH-LTB4	0.29±0.12	0.45±0.13	0.33±0.18	0.3±0.11	0.24±0.07	0.29±0.13	0.3±0.14	0.29±0.17	0.82±0.81
15-deoxy-PGJ2	0.42±0.19	0.29±0.15	0.48±0.24	0.5±0.14	0.53±0.23[a]	0.39±0.13[ab]	0.42±0.22[ab]	0.27±0.18[b]	3.73±5.51
20-OH-LTB4	ND	ND	ND	ND	ND	ND	ND	ND	ND
6-keto-PGF1a	ND	ND	ND	ND	ND	ND	ND	ND	ND
6-trans-LTB4	ND	ND	ND	ND	ND	ND	ND	ND	ND
LTB4	ND	ND	ND	ND	ND	ND	ND	ND	ND
LTC4	ND	ND	ND	ND	ND	ND	ND	ND	ND
LTD4	ND	ND	ND	ND	ND	ND	ND	ND	ND
LTE4	ND	ND	ND	ND	ND	ND	ND	ND	ND
LXA4	1.6±0.76	1.11±0.76	1.44±0.59	1.27±0.72	0.91±0.4	0.74±0.4	0.77±0.33	0.85±0.65	2.96$^{n=2}$
PGB2	1.32±0.71	1.95±1.04	1.07±0.57	1.58±0.6	1.55±0.78	1.21±0.43	1.03±0.34	10.01±21.5	1.69$^{n=2}$
PGD2	ND	ND	ND	ND	ND	ND	ND	ND	ND
PGE2	0.03±0.02$^{n=4}$	0.1±0.05$^{n=4}$	0.31$^{n=2}$	0.04±0.03$^{n=4}$	0.02±0.02	0.03±0.02	0.05±0.07	0.03±0.04	0.08$^{n=1}$
PGF2a	4.58±1.52	5.04±2.41	4.76±3.18	5.73±1.67	4.63±2.13	4.13±1.77	3.91±1.84	6.76±3.98	6.83±5.85
PGJ2	ND	ND	ND	ND	ND	ND	ND	ND	ND
TXB2	ND	ND	ND	ND	ND	ND	ND	ND	ND
**ALA-derived oxylipins**									
13-HOTrE	4.17±3.27[ab]	3.05±2.91[ab]	2.08±1.27[a]	8.67±8.87[b]	2.11±1.28	2.62±1.45	3.96±3.1	3.29±3.19	1.39$^{n=2}$
9-HOTrE	4.92±7.03	4.79±7.42	1.97±2.03	13.07±16.2	1.09±0.38	2.59±2.54	2.94±4.46	2.88±3.27	1.46$^{n=2}$
**EPA-derived oxylipins**									
15-HEPE	6.64±1.91	5.94±2.46	5.63±1.41	8.29±6.33	7.94±6.05	4.91±1.89	7.51±4.64	36.28±72.86	3.95±1.9
12-HEPE	ND	ND	ND	ND	ND	ND	ND	ND	ND
8-HEPE	ND	ND	ND	ND	ND	ND	ND	ND	ND
5-HEPE	2.31±0.46	2.55±1.69	2.81±1.84	2.2±0.33	1.73±0.54	1.77±0.74	2.2±0.36	4.81±7.46	1.62$^{n=2}$
17(18)-EpETE	9.32±1.48[a]	5.36±2.63[b]	6.83±4.48[ab]	6.43±2.45[ab]	5.82±1.04$^{n=5}$	5.37$^{n=2}$	5.21±1.98$^{n=6}$	6.99±5.22	ND

14(15)-EpETE	0.17±0.06	0.2±0.07	0.17±0.09	0.2±0.19	0.16±0.06	0.14±0.08	0.19±0.12	0.19±0.17	ND
11(12)-EpETE	0.88±0.08^{ab}	0.68±0.16^{c}	0.95±0.20^{a}	0.76±0.17^{bc}	0.73±0.1	0.69±0.23	0.74±0.15	0.59±0.16	0.08$^{n=1}$
8(9)-EpETE	1.91±0.9	1.67±0.58	1.82±1.03	1.58±0.54	1.66±0.78	1.61±1.07	2.02±0.62	1.7±0.85	0.35$^{n=1}$
17,18-DiHETE	ND	ND	ND	ND	ND	ND	ND	ND	ND
14,15-DiHETE	ND	ND	ND	ND	ND	ND	ND	ND	ND
11,12-DiHETE	ND	ND	ND	ND	ND	ND	ND	ND	ND
8,9-DiHETE	ND	ND	ND	ND	ND	ND	ND	ND	ND
5,6-DiHETE	ND	ND	ND	ND	ND	ND	ND	ND	ND
PGD3	ND	ND	ND	ND	ND	ND	ND	ND	ND
PGE3	ND	ND	ND	ND	ND	ND	ND	ND	ND
Resolvin E1	49.45±8.74	52.86±9.84	44.92±6.57	46.1±10.65	42.69±13.19	37.25±8.42	47.03±8.17	38.48±6.97	55.32±18.06
**DHA-derived oxylipins**									
17-HDoHE	21.26±5.84	19.5±8.42	23.5±5.21	23.39±8.7	17.89±3.19	18.27±9.51	20.04±8.77	17.43±3.1	ND
19(20)-EpDPE	174.79±26.54^{a}	118.51±43.03^{b}	152.52±50.36^{ab}	125.14±31.35^{b}	110.99±25.43	119.08±66.2	120.14±37.13	100.93±31.12	8.74±13.32
16(17)-EpDPE	95.17±14.28^{a}	65.10±26.87^{b}	81.74±26.56^{ab}	68.92±17.65^{b}	60.07±11.45	65.66±38.53	63.49±20.82	58.45±18.14	0.73$^{n=2}$
13(14)-EpDPE	101.02±15.07^{a}	66.1±27.93^{b}	84.49±25.09^{ab}	73.41±15.83^{b}	63.3±14.38	69.59±41.06	70.04±23.57	57.74±18.92	3.85$^{n=2}$
10(11)-EpDPE	81.03±12.78^{a}	57.17±27.8^{b}	66.81±11.15^{ab}	60.87±10.18^{ab}	51.95±16.42	56.65±32.53	58.5±18.09	45.97±14.32	2.84±3.73
7(8)-EpDPE	35.31±5.86^{a}	24.86±9.48^{b}	27.57±4.76^{b}	25.34±4.53^{b}	21.79±6.42	23.4±14.16	23.7±7.34	20.25±6.08	ND
19,20-DiHDPA	2.12±0.20^{a}	1.28±0.56^{b}	1.65±0.6^{ab}	1.23±0.37^{b}	1.48±0.33	1.51±0.7	1.47±0.41	1.31±0.7	0.12$^{n=1}$
16,17-DiHDPA	0.87±0.15^{a}	0.67±0.21^{ab}	0.74±0.3^{ab}	0.60±0.16^{b}	0.55±0.18	0.39±0.25	0.62±0.27	0.55±0.2	0.14$^{n=1}$

ND: not detected.

Different letters within a row of female or male group (highlighted values) are significantly different by one-way ANOVA followed by Duncan's post-hoc test ($p < 0.05$).

Data are expressed as mean$^{n=\text{detected number}}$(n<3) or mean ± SD $^{n=\text{detected number}}$(n≥3) if more than 3 values are missing in one group. Female data are described in the main text. In males, no significant differences in brain NL-bound oxylipins of LA, DGLA, ALA, EPA and DHA were observed; however, a few AA-derived oxylipins were altered (Supplementary Table 4). 9-HETE was 40% lower in Tg-TRAP versus Tg-FA rats ($p < 0.05$), 8-HETE was lower by 45% in Tg-TRAP compared to WT-TRAP, and 15-deoxy-PGJ2 was 49% lower in Tg-TRAP than WT-FA ($p < 0.05$).

Supplementary Table 5. Three-way ANOVA *p* value results of brain oxylipins in phospholipids (PLs) of 15-month old rats (n=54)

**Oxylipins**	**Source of variation**						
	**Sex**	**Exposure**	**Genotype**	**Sex × Exposure**	**Sex × Genotype**	**Exposure × Genotype**	**Sex × Exposure × Genotype**
**LA-derived oxylipins**							
13-HODE	ns	ns	ns	ns	ns	ns	ns
9-HODE	ns	ns	ns	ns	ns	ns	ns
13-oxo-ODE	0.0202	ns	ns	ns	0.0053	ns	ns
9-oxo-ODE	ns	ns	ns	ns	0.0037	ns	ns
12(13)-EpOME	0.0339	ns	ns	ns	ns	ns	ns
9(10)-EpOME	ns	ns	ns	ns	ns	ns	ns
12,13-DiHOME	ns	ns	ns	ns	ns	ns	ns
9,10-DiHOME	ns	ns	ns	ns	ns	ns	ns
9,12,13-TriHOME	ns	ns	ns	ns	ns	ns	ns
9,10,13-TriHOME	ns	ns	ns	ns	ns	ns	ns
**DGLA-derived oxylipins**							
15(S)-HETrE	ns	ns	ns	ns	ns	ns	ns
**AA-derived oxylipins**							
20-HETE	ns	ns	0.0407	ns	ns	ns	ns
15-HETE	ns	ns	ns	ns	0.0474	ns	ns
12-HETE	ns	ns	ns	ns	ns	ns	ns
11-HETE	ns	ns	ns	ns	0.0482	ns	ns
9-HETE	ns	ns	ns	ns	0.0256	ns	ns
8-HETE	ns	ns	0.0127	ns	ns	ns	ns
5-HETE	ns	ns	ns	ns	ns	ns	ns
15-oxo-ETE	ns	ns	ns	ns	0.0142	ns	ns
12-oxo-ETE	ns	ns	ns	ns	0.0446	0.0189	ns
14(15)-EpETrE	ns	ns	ns	ns	ns	ns	ns
8(9)-EpETrE	ns	ns	0.0288	ns	ns	ns	ns
5(6)-EpETrE	ns	ns	ns	ns	ns	ns	ns
14,15-DiHETrE	ns	ns	0.0113	ns	0.0201	ns	ns
11,12-DiHETrE	ns	0.0369	0.0143	ns	ns	ns	ns
8,9-DiHETrE	ns	ns	0.0118	ns	ns	ns	ns

5,6-DiHETrE	ns	ns	ns	ns	0.0129	ns	ns
20-COOH-LTB4	ns	ns	ns	ns	ns	ns	ns
15-deoxy-PGJ2	ns	ns	ns	ns	ns	ns	ns
LXA4	ns	0.0409	ns	ns	ns	ns	ns
PGB2	ns	ns	ns	ns	ns	ns	ns
PGF2a	ns	ns	ns	ns	ns	ns	ns
**ALA-derived oxylipins**							
13-HOTrE	ns	ns	ns	ns	0.0088	ns	ns
9-HOTrE	ns	ns	ns	ns	ns	ns	ns
**EPA-derived oxylipins**							
15-HEPE	ns	ns	ns	ns	ns	ns	ns
5-HEPE	ns	ns	ns	ns	ns	ns	ns
17(18)-EpETE	ns	ns	ns	ns	ns	ns	ns
11(12)-EpETE	ns	ns	ns	ns	ns	ns	ns
8(9)-EpETE	ns	ns	ns	ns	0.0449	ns	ns
Resolvin E1	ns	ns	ns	ns	ns	ns	ns
**DHA-derived oxylipins**							
17-HDoHE	ns	ns	ns	ns	ns	ns	ns
19(20)-EpDPE	ns	ns	ns	ns	ns	ns	ns
16(17)-EpDPE	ns	ns	ns	ns	ns	ns	ns
13(14)-EpDPE	ns	ns	ns	ns	ns	ns	ns
10(11)-EpDPE	ns	ns	ns	ns	ns	ns	ns
19,20-DiHDPA	ns	ns	0.0131	ns	ns	ns	ns
16,17-DiHDPA	ns	0.0248	ns	ns	ns	ns	ns

ns: not significant, $p \geq 0.05$.

Sex significantly altered LA-derived 13-oxo-ODE and 12(13)-EpOME, which was higher by 21% and 19% in brain PLs of females than males, respectively ($p < 0.05$). Exposure effects were significant in the brain AA-derived 11,12-DiHETrE and LXA4, and DHA-derived 19,20-DiHDPA ($p < 0.05$). Genotype effects were significant in the brain AA-derived 20-HETE, 8-HETE, 8(9)-EpETrE, 14,15-DiHETrE, 11,12-DiHETrE and 8,9-DiHETrE, and DHA-derived 19,20-DiHDPA ($p < 0.05$).

Significant sex and genotype interaction effects were observed in LA-derived 13-oxo-ODE and 9-oxo-ODE, AA-derived 15-HETE, 11-HETE, 9-HETE, 15-oxo-ETE, 12-oxo-ETE, 14,15-DiHETrE and 5,6-DiHETrE, ALA-derived 13-HOTrE, and EPA-derived 8(9)-EpETE of brain PLs ($p < 0.05$). Significant exposure and genotype interaction effects were observed in AA-derived 12-oxo-ETE of brain PLs ($p < 0.05$).

Supplementary Table 6. Oxylipin concentrations in brain phospholipids (PLs) of 15-month old rats (n=54). A one-way ANOVA followed by Duncan's post-hoc test was applied per sex, to test for significant differences between wildtype (WT) and transgenic (Tg) rats exposed to filtered air (FA) or traffic-related air pollution (TRAP). Data are expressed as mean ± SD. WT: wildtype rats; Tg: Alzheimer's Disease transgenic rats; TRAP: traffic-related air pollution; FA: filtered air.

**Oxylipins (pmol/g)**	**Female (n=27)**				**Male (n=27)**				**Blank (n=3)**
	**WT-FA (n=7)**	**Tg-FA (n=7)**	**WT-TRAP (n=6)**	**Tg-TRAP (n=7)**	**WT-FA (n=7)**	**Tg-FA (n=7)**	**WT-TRAP (n=7)**	**Tg-TRAP (n=6)**	
**LA-derived oxylipins**									
13-HODE	235.5±459.92	40.96±8.27	52.15±17.18	44.87±8.92	46.41±17.5	49.85±10.74	35.99±7.34	44.81±18.56	17.45±1.04
9-HODE	133.13±282.63	15.77±4.48	22.12±7.2	18.55±5.77	18.69±8.36	19.27±5.13	14.31±4.84	19.09±9.04	6.75±1.83
13-oxo-ODE	36.28±4.84^{a}	25.00±8.93^{b}	31.68±11.10^{ab}	27.49±6.81^{ab}	21.61±3.96	28.24±10.78	23.63±4.81	26.46±8.53	12.85$^{n=2}$
9-oxo-ODE	22.59±4.50^{a}	13.62±4.48^{b}	19.67±7.75^{a}	12.78±2.89^{b}	12.75±3.45	14.7±6.25	15.4±3.47	17.31±11.24	1.75±0.75
12(13)-EpOME	30.58±7.74	24.01±5.34	28.9±9.02	23.49±4.44	23.68±11.49	21.2±4.2	21.33±5.9	23.2±8.15	1.50$^{n=2}$
9(10)-EpOME	7.15±0.98^{a}	4.73±1.85^{b}	6.21±2.68^{ab}	4.97±1.75^{ab}	5.25±2.25	4.97±0.93	5.06±1.52	5.43±2.21	1.89±1.62
12,13-DiHOME	3.29±1.04	3.19±1.14	3.39±0.55	3.26±0.6	3.96±1.1	3.24±0.73	3.06±0.7	3.07±0.89	3.86±0.08
9,10-DiHOME	5.43±1.51	5.37±2.8	4.56±0.61	5.37±1.57	5.5±1.54	4.24±1.05	5.26±1.49	4.77±2.07	5.02±0.99
9,12,13-TriHOME	101.58±69.78	96.89±69.15	70.34±17.37	86.86±47.35	88.37±45.65	94.96±53.31	80.56±41.35	140.89±197.48	75.13±18.55
9,10,13-TriHOME	26.75±17.73	24.75±22.76	16.07±5.11	22.2±13.36	22.65±13.76	22.8±12.2	18.28±10.51	36.42±50.8	22.63±8.99
**DGLA-derived oxylipins**									
15(S)-HETrE	8.37±7.85	3.53±1.16	4.21±1.03	3.66±0.57	3.96±1.67	3.63±1.86	3.57±0.52	3.92±2.06	0.35±0.06
LTB3	ND	ND	ND	ND	ND	ND	ND	ND	ND
PGD1	ND	ND	ND	ND	ND	ND	ND	ND	ND
PGE1	ND	ND	ND	ND	ND	ND	ND	ND	ND
**AA-derived oxylipins**									
20-HETE	113.92±31.52	78.23±25.21	95.76±40.64	84.08±16.74	98.63±31.8	74.36±26.23	87.82±26.24	91.92±33.81	83.91±31.56
15-HETE	60.32±21.04^{a}	28.24±15.54^{b}	38.14±14.08^{b}	28.50±6.08^{b}	40.43±23.94	35.51±24.32	33.59±13.74	39.65±27.07	1.74$^{n=2}$
12-HETE	12.06±5.00^{a}	7.00±3.61^{b}	8.87±3.67^{ab}	7.63±1.90^{b}	7.94±3.71	8.39±5.5	7.89±2.14	7.99±4.25	0.08$^{n=2}$
11-HETE	25.34±11.07^{a}	12.16±5.01^{b}	16.25±5.63^{b}	12.5±1.89^{b}	16.39±8.59	14.86±9.04	14.76±5.17	16.37±10.54	0.8±0.16
9-HETE	50.03±26.89^{a}	21.66±10.78^{b}	29.92±10.47^{b}	22.9±4.24^{b}	27.4±13.45	27.1±20.32	24.73±7.29	28.81±17.65	0.86±0.72
8-HETE	11.21±5.14^{a}	4.90±2.68^{b}	7.40±3.41^{ab}	5.87±0.90^{b}	6.51±2.9	5.5±3.59	7.17±2.09	6.65±4.11	ND
5-HETE	76.25±26.26^{a}	39.38±19.40^{b}	52.36±20.04^{b}	41.79±8.68^{b}	49.4±27.88	46.7±35.16	43.97±13.3	49.81±27.8	1.50$^{n=2}$
15-oxo-ETE	14.69±3.32^{a}	8.13±3.52^{b}	11.97±3.92^{ab}	9.31±2.35^{b}	10.15±3.48	10.12±4.61	10.7±2.07	12.54±7.05	0.59±0.2

12-oxo-ETE	5.52±1.90^{a}	2.94±1.53^{b}	4.11±1.09^{ab}	4.10±1.76^{ab}	3.42±1.36	3.06±0.97	3.42±0.5	4.94±3.17	1.35$^{n=2}$
5-oxo-ETE	ND	ND	ND	ND	ND	ND	ND	ND	ND
8,15-DiHETE	ND	ND	ND	ND	ND	ND	ND	ND	ND
5,15-DiHETE	1.96±1.25$^{n=5}$	1.08$^{n=2}$	1.07±0.36$^{n=5}$	1.05±0.34$^{n=4}$	1.3±0.48$^{n=3}$	1.06$^{n=2}$	1.24±0.28$^{n=3}$	1.10$^{n=2}$	ND
14(15)-EpETrE	150.69±39.42	121.88±27.95	143.73±41.08	117.87±33.49	152.07±80.58	133.58±42.54	113.51±41.32	134.92±40.12	4.68±4.39
11(12)-EpETrE	0.37±0.33	2.19±3.14	0.52±0.34	0.6±1.04	1.41±1.39$^{n=6}$	3.94±2.65$^{n=4}$	0.23$^{n=1}$	1.1±1.12$^{n=4}$	1.82±1.32
8(9)-EpETrE	73.70±13.98^{a}	54.04±8.05^{b}	63.00±20.46^{ab}	57.2±10.30^{b}	63.21±24.47	52.39±15.15	53.78±9.37	53.21±10.1	1.86±1.99
5(6)-EpETrE	11.06±2.48	7.74±5.27	10.21±5.56	6.8±3.51	9.29±5.63	8.45±3.91	7.5±2.79	9.25±5.14	0.32±0.12
14,15-DiHETrE	2.5±0.73^{a}	1.25±0.59^{b}	1.82±0.47^{b}	1.19±0.46^{b}	1.88±0.74	1.57±0.89	1.5±0.31	1.72±1.03	0.31$^{n=1}$
11,12-DiHETrE	1.63±0.38^{a}	1.05±0.46^{b}	1.11±0.30^{b}	0.81±0.37^{b}	1.26±0.92	1.01±0.43	1.05±0.3	0.86±0.27	0.27$^{n=1}$
8,9-DiHETrE	2.50±1.12^{a}	1.23±0.35^{b}	2.24±1.39^{ab}	1.38±0.39^{b}	2.18±1.45	1.92±1.27	1.82±0.84	1.35±0.29	1.05$^{n=2}$
5,6-DiHETrE	36.9±10.23^{a}	21.45±5.55^{b}	28.47±7.35^{b}	21.58±4.44^{b}	27.01±12.28	26.24±12.57	24.04±8.12	29.7±12.91	0.97$^{n=2}$
20-COOH-LTB4	0.26±0.2	0.15±0.13	0.16±0.16	0.16±0.12	0.17±0.14	0.08±0.09	0.19±0.12	0.11±0.09	0.17±0.14
15-deoxy-PGJ2	0.54±0.43	0.48±0.47	0.47±0.28	0.51±0.18	0.48±0.27	3.3±7.25	0.44±0.28	0.44±0.23	2.64±0.51
20-OH-LTB4	ND	ND	ND	ND	ND	ND	ND	ND	ND
6-keto-PGF1a	ND	ND	ND	ND	ND	ND	ND	ND	ND
6-trans-LTB4	ND	ND	ND	ND	ND	ND	ND	ND	ND
LTB4	ND	ND	ND	ND	ND	ND	ND	ND	ND
LTC4	ND	ND	ND	ND	ND	ND	ND	ND	ND
LTD4	ND	ND	ND	ND	ND	ND	ND	ND	ND
LTE4	ND	ND	ND	ND	ND	ND	ND	ND	ND
LXA4	8.4±1.9	7.61±1.69	7.34±1.63	6.96±1.96	9.06±5.01	8±2.83	5.93±2.94	6.66±1.23	9.57±3.21
PGB2	7.68±4.71^{a}	2.83±3.97^{b}	4.69±3.23^{ab}	2.94±1.94^{b}	4.13±4.95	4.06±5.2	4.45±4.76	4.57±3.5	0.10$^{n=1}$
PGD2	ND	ND	ND	ND	ND	ND	ND	ND	ND
PGE2	0.42±0.24^{a}	0.16±0.13^{b}	0.4±0.23^{ab}	0.26±0.23^{ab}	0.42±0.14$^{n=3}$	0.29±0.21$^{n=5}$	0.53$^{n=2}$	0.51±0.55$^{n=4}$	ND
PGF2a	23.86±11.36	24.03±7.37	17.4±5.53	20.9±7.04	22.65±14.2	21.68±7.47	20.59±6.69	23.45±5.43	17.21±2.97
PGJ2	ND	ND	ND	ND	ND	ND	ND	ND	ND
TXB2	ND	ND	ND	ND	ND	ND	ND	ND	ND
**ALA-derived oxylipins**									
13-HOTrE	10.06±6.76	5.59±1.25	6.7±2.47	5.38±1.99	6.1±3.16	7.17±2.35	4.73±1.94	7.58±2.57	2.24±0.59
9-HOTrE	4.01±5.19	2.21±1.23	1.68±0.44	2.22±0.88	1.99±0.83	2.12±1.48	2.04±0.59	1.45±0.71	3.22±0.46
**EPA-derived oxylipins**									
15-HEPE	173.38±50.75^{a}	100.91±28.19^{b}	126.41±39.98^{b}	103.2±25.62^{b}	127.6±63.24	135.87±103.68	96.45±26.82	112.41±56.47	34.58±12.22
12-HEPE	ND	ND	ND	ND	ND	ND	ND	ND	ND
8-HEPE	ND	ND	ND	ND	ND	ND	ND	ND	ND

5-HEPE	11.16±11.36	7.57±4.78	7.49±3.53	7.68±3.01	5.25±1.68	32.61±74.18	6±1.27	5.27±3.04	2.55±1.19
17(18)-EpETE	22.35±9.47	16.9±10.58	23.73±14.3	19.83±13.26	23.4±19.18	23.84±13.09	19.97±14	30.45±21.34	7.16±3.27
14(15)-EpETE	0.20±0.12	0.20±0.15$^{n=3}$	0.20±0.10	0.11±0.13$^{n=4}$	0.18±0.05	0.4±0.63	0.18±0.09	0.36±0.27	0.10$^{n=2}$
11(12)-EpETE	5.2±0.95	3.95±1.85	4.66±1.71	3.8±0.91	4.18±1.89	3.67±2.13	4.03±0.97	4.65±2.41	0.23$^{n=2}$
8(9)-EpETE	6.1±3	4.06±2.99	6.4±1.97	4.74±3.81	5.07±1.89	6.19±4	5.05±1.65	6.85±3.24	0.56$^{n=1}$
17,18-DiHETE	ND	ND	ND	ND	ND	ND	ND	ND	ND
14,15-DiHETE	ND	ND	ND	ND	ND	ND	ND	ND	ND
11,12-DiHETE	ND	ND	ND	ND	ND	ND	ND	ND	ND
8,9-DiHETE	ND	ND	ND	ND	ND	ND	ND	ND	ND
5,6-DiHETE	9.13±3.83$^{n=4}$	3.05$^{n=1}$	5.60$^{n=2}$	3.71$^{n=1}$	4.89$^{n=2}$	7.68$^{n=2}$	5.45$^{n=2}$	5.32±1.39$^{n=4}$	ND
PGD3	ND	ND	ND	ND	ND	ND	ND	ND	ND
PGE3	ND	ND	ND	ND	ND	ND	ND	ND	ND
Resolvin E1	54.73±23.85	55.57±18.84	51.11±14.32	48.94±12.08	53.39±34.78	50.96±8.02	41.25±8.77	45.05±9.73	83.55±20.01
**DHA-derived oxylipins**									
17-HDoHE	81.61±14.54^{a}	47.31±19.51^{b}	56.38±18^{b}	44.24±5.58^{b}	57.43±28.24	54.22±27.27	54±20.3	56.63±32.32	2.68±0.52
19(20)-EpDPE	290.37±47.74^{a}	219.43±60.18^{b}	270.49±73.87^{ab}	215.04±48.66^{b}	233.57±112.9	220.96±61.75	196.54±66.55	234±79.11	4.54$^{n=2}$
16(17)-EpDPE	76.92±16.96	55.42±25.19	69±22.38	52.32±20.63	63.35±34.17	59.06±18.39	51.39±18.8	61.12±19.23	2.36±1.62
13(14)-EpDPE	22.1±3.89	15.98±10.34	21.35±10.82	14.02±6.79	19.01±12.18	16.97±6.68	14.67±7.35	18.95±7.72	2.07±1.34
10(11)-EpDPE	0.72±0.32	0.55±0.43	0.79±0.51	0.47±0.35	0.55±0.69	0.48±0.32	0.41±0.41	0.69±0.54	1.99±1.3
7 (8)-EpDPE	1.72±0.56	4.34±4.35	1.54±1.04	1.83±1.5	3.49±3.51$^{n=4}$	6.38±4.94$^{n=4}$	0.78$^{n=2}$	2.27±1.18$^{n=3}$	ND
19,20-DiHDPA	5.08±1.61^{a}	2.96±0.73^{b}	3.62±1.05^{b}	2.54±0.6^{b}	3.4±1.75	3.09±1.88	3.12±0.87	2.9±1.39	0.17±0.28
16,17-DiHDPA	2.31±0.68^{a}	1.54±0.48^{b}	1.45±0.52^{b}	1.23±0.33^{b}	1.68±0.92	1.45±0.59	1.34±0.45	1.43±0.59	0.18$^{n=1}$

ND: not detected.

Different letters within a row of female or male group (highlighted values) are significantly different by one-way ANOVA followed by Duncan's post-hoc test ($p < 0.05$).

Data are expressed as mean$^{n=\text{detected number}}$(n<3) or mean ± SD $^{n=\text{detected number}}$(n≥3) if mor than 3 values are missing in one group.

Supplementary Table 7. Multiple linear regression analysis relating log-transformed oxylipin concentrations in phospholipids of human prefrontal cortex (dependent variable), to diagnosis group (AD/non-AD), sex, mean-centered postmortem interval (PMI) and mean centered age were tested for main effects (n=41). Interaction effects were tested between group and age, and between group and sex (n=41).

**Oxylipins**	**Variable**	**Estimate**	**Standard error**	**95% CI (asymptotic)**	**|t|**	**P value**	**P value summary**
14(15)-EpETrE	Intercept	3.876	0.1321	3.608 to 4.145	29.34	<0.0001	****
	Group[1][a]	-0.2332	0.1832	-0.6055 to 0.1390	1.273	0.2116	ns
	Sex[0][b]	-0.03433	0.1883	-0.4171 to 0.3484	0.1823	0.8564	ns
	PMI	-0.002310	0.002593	-0.007580 to 0.002960	0.8906	0.3794	ns
	Age	0.03538	0.01138	0.01225 to 0.05850	3.109	0.0038	**
	Group[1] : Sex[0]	0.09468	0.2643	-0.4424 to 0.6318	0.3582	0.7224	ns
	Group[1] : Age	-0.04709	0.01788	-0.08344 to -0.01074	2.633	0.0126	*
19(20)-EpDPE	Intercept	3.580	0.2762	3.019 to 4.142	12.96	<0.0001	****
	Group[1]	-0.6174	0.3830	-1.396 to 0.1609	1.612	0.1162	ns
	Sex[0]	-0.5107	0.3937	-1.311 to 0.2894	1.297	0.2033	ns
	PMI	-0.003228	0.005422	-0.01425 to 0.007791	0.5953	0.5556	ns
	Age	0.04831	0.02379	-2.479e-005 to 0.09665	2.031	0.0501	ns
	Group[1] : Sex[0]	0.3938	0.5526	-0.7291 to 1.517	0.7127	0.4809	ns
	Group[1] : Age	-0.08187	0.03739	-0.1579 to -0.005881	2.190	0.0355	*
13-HODE	Intercept	0.7601	0.07022	0.6174 to 0.9028	10.82	<0.0001	****
	Group[1]	0.003451	0.09737	-0.1944 to 0.2013	0.03544	0.9719	ns
	Sex[0]	0.03957	0.1001	-0.1639 to 0.2430	0.3953	0.6951	ns
	PMI	-0.0009166	0.001378	-0.003718 to 0.001885	0.6650	0.5106	ns
	Age	-0.008631	0.006047	-0.02092 to 0.003658	1.427	0.1626	ns

	Group[1] : Sex[0]	0.01676	0.1405	-0.2687 to 0.3023	0.1193	0.9058	ns
	Group[1] : Age	0.02305	0.009507	0.003733 to 0.04237	2.425	0.0208	*
17-HDoHE	Intercept	1.972	0.1069	1.754 to 2.189	18.44	<0.0001	****
	Group[1]	-0.1933	0.1482	-0.4946 to 0.1080	1.304	0.2010	ns
	Sex[0]	-0.1272	0.1524	-0.4369 to 0.1825	0.8347	0.4097	ns
	PMI	-0.0009145	0.002099	-0.005180 to 0.003351	0.4357	0.6658	ns
	Age	-0.01301	0.009207	-0.03172 to 0.005703	1.413	0.1668	ns
	Group[1] : Sex[0]	0.3551	0.2139	-0.07962 to 0.7898	1.660	0.1061	ns
	Group[1] : Age	0.04075	0.01447	0.01133 to 0.07016	2.815	0.0081	**
9-HODE	Intercept	0.7592	0.06873	0.6196 to 0.8989	11.05	<0.0001	****
	Group[1]	0.01301	0.09530	-0.1807 to 0.2067	0.1366	0.8922	ns
	Sex[0]	0.03930	0.09798	-0.1598 to 0.2384	0.4011	0.6909	ns
	PMI	-0.0006862	0.001349	-0.003428 to 0.002056	0.5086	0.6143	ns
	Age	-0.004720	0.005919	-0.01675 to 0.007309	0.7974	0.4307	ns
	Group[1] : Sex[0]	-0.01656	0.1375	-0.2960 to 0.2629	0.1205	0.9048	ns
	Group[1] : Age	0.02411	0.009305	0.005203 to 0.04302	2.591	0.0140	*
11-HETE	Intercept	1.817	0.1032	1.607 to 2.027	17.60	<0.0001	****
	Group[1]	-0.1217	0.1432	-0.4127 to 0.1692	0.8504	0.4010	ns
	Sex[0]	-0.03080	0.1472	-0.3299 to 0.2683	0.2092	0.8355	ns
	PMI	-0.001742	0.002027	-0.005861 to 0.002376	0.8597	0.3960	ns
	Age	-0.01552	0.008891	-0.03359 to 0.002552	1.745	0.0900	ns
	Group[1] : Sex[0]	0.1613	0.2066	-0.2585 to 0.5811	0.7808	0.4403	ns

	Group[1] : Age	0.03402	0.01398	0.005615 to 0.06243	2.434	0.0203	*
12-HETE	Intercept	1.777	0.1032	1.567 to 1.987	17.21	<0.0001	****
	Group[1]	-0.08717	0.1432	-0.3781 to 0.2038	0.6089	0.5466	ns
	Sex[0]	-0.03108	0.1472	-0.3302 to 0.2680	0.2112	0.8340	ns
	PMI	-0.001028	0.002027	-0.005147 to 0.003090	0.5074	0.6151	ns
	Age	-0.01542	0.008891	-0.03349 to 0.002646	1.735	0.0919	ns
	Group[1] : Sex[0]	0.09475	0.2066	-0.3250 to 0.5145	0.4587	0.6493	ns
	Group[1] : Age	0.03641	0.01398	0.008006 to 0.06482	2.605	0.0135	*
15-HETE	Intercept	2.251	0.1220	2.004 to 2.499	18.46	<0.0001	****
	Group[1]	-0.1196	0.1691	-0.4633 to 0.2241	0.7073	0.4842	ns
	Sex[0]	-0.01668	0.1739	-0.3700 to 0.3367	0.09592	0.9241	ns
	PMI	-0.002107	0.002394	-0.006973 to 0.002759	0.8801	0.3850	ns
	Age	-0.02013	0.01050	-0.04148 to 0.001217	1.916	0.0638	ns
	Group[1] : Sex[0]	0.1661	0.2440	-0.3298 to 0.6620	0.6807	0.5007	ns
	Group[1] : Age	0.04444	0.01651	0.01088 to 0.07800	2.691	0.0110	*
15(S)-HETrE	Intercept	1.372	0.1053	1.158 to 1.586	13.03	<0.0001	****
	Group[1]	-0.07948	0.1460	-0.3762 to 0.2173	0.5443	0.5898	ns
	Sex[0]	-0.01172	0.1501	-0.3168 to 0.2934	0.07807	0.9382	ns
	PMI	-0.001131	0.002067	-0.005332 to 0.003070	0.5469	0.5880	ns
	Age	-0.01881	0.009069	-0.03724 to -0.0003756	2.074	0.0458	*
	Group[1] : Sex[0]	0.1231	0.2107	-0.3050 to 0.5513	0.5845	0.5627	ns
	Group[1] : Age	0.03606	0.01426	0.007082 to 0.06503	2.529	0.0162	*
5-HETE	Intercept	2.259	0.1119	2.032 to 2.486	20.19	<0.0001	****
	Group[1]	-0.07396	0.1552	-0.3893 to 0.2414	0.4766	0.6367	ns
	Sex[0]	0.03943	0.1595	-0.2848 to 0.3636	0.2471	0.8063	ns

	PMI	-0.0008013	0.002197	-0.005266 to 0.003663	0.3648	0.7175	ns
	Age	-0.02243	0.009637	-0.04201 to -0.002841	2.327	0.0261	*
	Group[1] : Sex[0]	0.1441	0.2239	-0.3109 to 0.5991	0.6435	0.5242	ns
	Group[1] : Age	0.04216	0.01515	0.01137 to 0.07295	2.783	0.0087	**
8-HETE	Intercept	1.795	0.08601	1.620 to 1.970	20.87	<0.0001	****
	Group[1]	-0.09229	0.1193	-0.3346 to 0.1501	0.7739	0.4444	ns
	Sex[0]	-0.03499	0.1226	-0.2842 to 0.2142	0.2854	0.7771	ns
	PMI	-0.0006821	0.001688	-0.004113 to 0.002749	0.4040	0.6887	ns
	Age	-0.01420	0.007407	-0.02926 to 0.0008482	1.918	0.0636	ns
	Group[1] : Sex[0]	0.1318	0.1721	-0.2179 to 0.4815	0.7659	0.4490	ns
	Group[1] : Age	0.03235	0.01164	0.008687 to 0.05601	2.778	0.0088	**

[a] Group 1 is the AD group; [b] 0 refers to males in the coding, and 1 is for females. ns, $p>0.05$; *, $p<0.05$; **, $p<0.01$, ***, $p<0.001$, ****, $p<0.0001$
